# Exploring health equity in Lesotho’s Child Grants Programme

**DOI:** 10.1093/heapol/czad116

**Published:** 2024-01-20

**Authors:** Elodie Besnier, Virginia Kotzias, Thandie Hlabana, Kathryn Beck, Céline Sieu, Kimanzi Muthengi

**Affiliations:** Centre for Global Health Inequalities Research (CHAIN), Department of Sociology and Political Science, Norwegian University of Science and Technology (NTNU), PO box 8900, Torgarden, Trondheim 7491, Norway; Department of Public Health and Nursing, Faculty of Medicine and Health Sciences, NTNU PO box 8900, Torgarden, Trondheim 7491, Norway; Centre for Global Health Inequalities Research (CHAIN), Department of Sociology and Political Science, Norwegian University of Science and Technology (NTNU), PO box 8900, Torgarden, Trondheim 7491, Norway; National University of Lesotho, P.O Roma 180, Roma, Lesotho; School of Environmental Sciences, University of Hull, Cottingham Rd, Hull HU6 7RX, United Kingdom; Centre for Global Health Inequalities Research (CHAIN), Department of Sociology and Political Science, Norwegian University of Science and Technology (NTNU), PO box 8900, Torgarden, Trondheim 7491, Norway; Centre for Fertility and Health, Norwegian Institute of Public Health, PO Box 222 Skøyen, Oslo N-0213, Norway; UNICEF Lesotho Country office, 13 UN Road UN House, Maseru, Lesotho; UNICEF Lesotho Country office, 13 UN Road UN House, Maseru, Lesotho

**Keywords:** Child health, cash transfer, Lesotho, health equity, economic empowerment, social protection

## Abstract

Despite their growing popularity, little is known about how cash transfers (CTs) can affect health equity in targeted communities. Lesotho’s Child Grants Programme (CGP) is an unconditional CT targeting poor and vulnerable households with children. Started in 2009, the CGP is one of Lesotho’s key programmes in developing the country’s social protection system. Using the CGP’s early phases as a case study, this research aims to capture how programme stakeholders understood and operationalized the concept of health equity in Lesotho’s CGP. The qualitative analysis relied on the triangulation of findings from a desk review and semi-structured key informant interviews with programme stakeholders. The programme documents were coded deductively and the interview transcripts inductively. Both materials were analysed thematically before triangulating their findings. We explored determining factors for differences or disagreements within a theme according to the programme’s chronology, the stakeholders’ affiliations and their role(s) in the CGP. The definitions of health equity in the context of the CGP reflected an awareness among stakeholders of these issues and their determinants but also the challenges raised by the complex (or even debated) nature of the concept. The most common definition of this concept focused on children’s access to health services for the most disadvantaged households, suggesting a narrow, targeted approach to health equity as targeting disadvantages. Yet, even the most common definition of this concept was not fully translated into the programme, especially in the day-to-day operations and reporting at the local level. This operationalization gap affected the study of selected health spillover effects of the CGP on health equity and might have undermined other programme impacts related to specific health disadvantages or gaps. As equity objectives become more prominent in CTs, understanding their meaning and translation into concrete, observable and measurable applications in programmes are essential to support impact.

Key messages

- The Child Grants Programme (CGP) had holistic and ambitious vision, which explicitly or implicitly touched upon many dimensions of child health equity.
- The definitions of health equity in the context of the CGP reflected an awareness among stakeholders of these issues and their determinants but also the challenges raised by the complex (or even debated) nature of the concept.
- Despite some consensus on how the concept was understood, there were wide variations in the operationalization of the different definitions of health equity.
- This operationalization gap affected the study of selected health equity effects of the CGP and might have undermined other programme health equity impacts.

## Introduction

Cash transfers (CTs) have been increasingly used in low- and middle-income countries as a social protection tool (Bastagli *et al.*, 2016). CTs are programmes providing non-contributory monetary grants to individuals (UNICEF-ESARO, Transfer Project, 2015). They are part of the African Union’s Social Policy Framework (African Union Commission, 2008), which identifies benefits for children (including health care) as part of the essential social protection package for African countries. The Social Policy Framework for Africa also links social protection to equitable access to health care and the reduction of inequalities between and within the countries of the region (African Union Commission, 2008). CTs are associated with the increased use of child health services and improvements in child health and nutrition while simultaneously addressing numerous social determinants of health for beneficiary households, including school attendance, asset ownership, social capital and empowerment (Lagarde *et al.*, 2009; Owusu-Addo and Cross, 2014; Bastagli *et al.*, 2016; Bonilla *et al.*, 2017; Pega *et al.*, 2017; Walque, 2017). This suggests that by addressing selected vulnerabilities in beneficiary children and their caregivers, CTs can contribute to promoting health equity. Evidence of CTs’ effects on local economies would also suggest that CTs might have effects beyond the beneficiary group (Taylor and Thome, 2016; Thome *et al.*, 2016; Gupta *et al.*, 2018). However, little is known about the consequences and implications of these programmes for health disparities among children (Owusu-Addo *et al.*, 2018; Besnier *et al.*, 2021).

In the context of high prevalence of HIV and AIDS and the resulting orphanhood and poverty, Lesotho’s Child Grants Programme (CGP) was started in 2009. It is an unconditional CT aiming to improve the living standards of orphans and vulnerable children (OVC) to reduce malnutrition, improve health status and increase school enrolment (Pellerano *et al.*, 2014a). In the 2014 evaluation, the CGP showed promising results regarding selected economic outcomes, child health outcomes and determinants of health amongst beneficiary households. However, the health and human development effects of the programme at the community level have not been studied. While the CGP’s theory of change^1^ highlighted the key aim of reducing inequalities (including in child health) (Pellerano *et al.*, 2014a), the definition and integration of this idea into the programme remain unclear.

The Empowerment for Health Equity—Lesotho (E4HE Lesotho) project is a mixed-methods case study intended to inform the study of health inequalities and power issues in CTs like the CGP. This article is the second of the E4HE Lesotho series and focuses on how the concept of health equity was perceived and operationalized (i.e. made concrete, observable and measurable in the programme) by CGP stakeholders in the early phases of the programme, to inform future CGP evaluation and development.

### Aim and research questions

This study aims to capture how programme stakeholders understood and operationalized ‘health equity’ in the early phases of Lesotho’s CGP, prior to the introduction of complementary or Cash Plus pilots. More specifically, this study aimed to answer the following questions:

- How do programme stakeholders define the concept of health equity?
- What role do they see this concept plays in the programme (e.g. in the programme cycle, design features and effects)?
- Did these roles and definitions evolve over time?
- What was the programme stakeholders’ perception of the effects of the CGP on child health equity in the treatment communities?

### Conceptual background: understanding health equity

**Figure 1.**
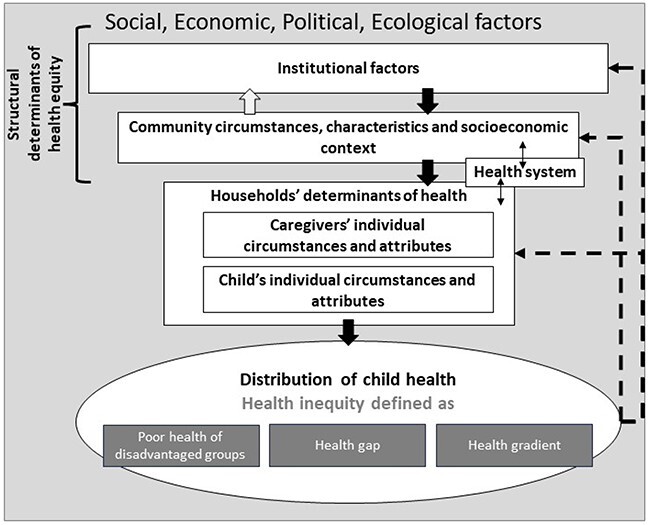
Understanding health equity: mapping elements of definitions

Health (in)equities can be defined as ‘avoidable’ health inequalities (Kawachi *et al.*, 2002; Whitehead and Dahlgren, 2006; Commission on Social Determinants of Health, 2008). Behind this concept is the idea that health and its distribution in a population are shaped by factors beyond our control, known as determinants of health. These determinants are shaped by wider, structural, social, political or economic factors that define their unequal distributions in society, shaping health (in)equity. Since young children are dependent on others for their health and development, child health is determined both by children’s individual specificities and by their caregivers’ circumstances (see the upper half of Figure 1) (Commission on Social Determinants of Health, 2008; Black *et al.*, 2017).

*Figure developed from Graham (2004) and Commission on Social Determinants of Health (2008).

Graham’s (2004) typology of interventions illustrates some of the different conceptions and definitions of health inequalities (see the bottom half of Figure 1) and how one’s understanding of this concept implies a specific approach to health equity. Hence, to understand how CT programmes might affect child health equity, it is essential to understand its conception(s) adopted in the programme and how it was turned into concrete, observable and measurable programme elements. Graham’s typology also shows how health equity is often used interchangeably with health inequalities (Whitehead and Dahlgren, 2006). Our use of the term in the E4HE case study is described in Supplementary Annexe 1. In this paper, we chose to reflect the exact terminology used by cited authors or by CGP stakeholders. At one end of Graham’s continuum, addressing the inequalities that threaten health equity involves targeted interventions focusing on the needs of a disadvantaged group to ensure a minimum health ‘level’. Under this scenario, a CT programme would lead to improvement amongst beneficiary children, thus reducing their health disadvantage but not necessarily the disparities between beneficiaries and other children in the community. Graham defines approaching health inequalities as the disparities in health between two groups as the ‘health gap’. Under this approach, a CT programme would make beneficiaries’ health improve quickly to catch-up to the better-off groups. At the end of the spectrum, health inequalities are defined as a gradient, directly related to the socioeconomic structure of a population. This scenario implies that a CT programme affects the wider community directly or indirectly and leads to structural changes that make the whole community healthier and more equity-driven.

## Method

This descriptive qualitative study used thematic analysis and data triangulation and is one of the qualitative components of the E4HE Lesotho mixed-methods case study. The rich narrative data yielded from this qualitative descriptive study enriched our understanding of health equity and laid a robust foundation for the quantitative components of the mixed-methods case study.

### Study setting

Lesotho has been categorized as a ‘least developed country’ since 1971 (UNCTAD, 2021). Tensions between political parties, a struggling economy and persistent social and gender inequalities have contributed to recurring political instability (Shale, 2021). When the CGP was introduced in 2009, more than half of Lesotho’s children lived in absolute poverty (i.e. deprived in two or more essential dimensions), with rates up to 80% in the mountain areas (UNICEF, 2011). Lesotho had the third highest HIV prevalence rate globally, fuelling mortality and orphanhood. These challenges were unevenly shared among Basotho communities, raising equity issues for children’s health (see Table 1) (Ministry of Health and Social Welfare—MOHSW/Lesotho, ICF Macro, 2010; UNICEF, 2011; UNFPA, 2012).

**Table 1.** Disparities across selected child health indicators in Lesotho^a^

	Residence		Wealth quintiles		Mother’s education	
	Urban	Rural	Highest	Lowest	Secondary	Did not finish primary school
Child mortality rate (per 1000 live births)	89	110	80	107	88	124
Vaccination coverage in children 12–23 months (in %)	71	59	72	52	66	54
Prevalence of acute respiratory infection in children under age 5 (in %)	2.7	6.3	1.8	7.9	4.3	7.2
Prevalence of diarrhoea in children under age 5 (in %)	9.8	11.6	8,6	13.8	9.7	12.9

a Source: Ministry of Health and Social Welfare—MOHSW/Lesotho, ICF Macro (2010). Lesotho Demographic and Health Survey 2009. MOHSW and ICF Macro, Maseru.

**Figure 2.**
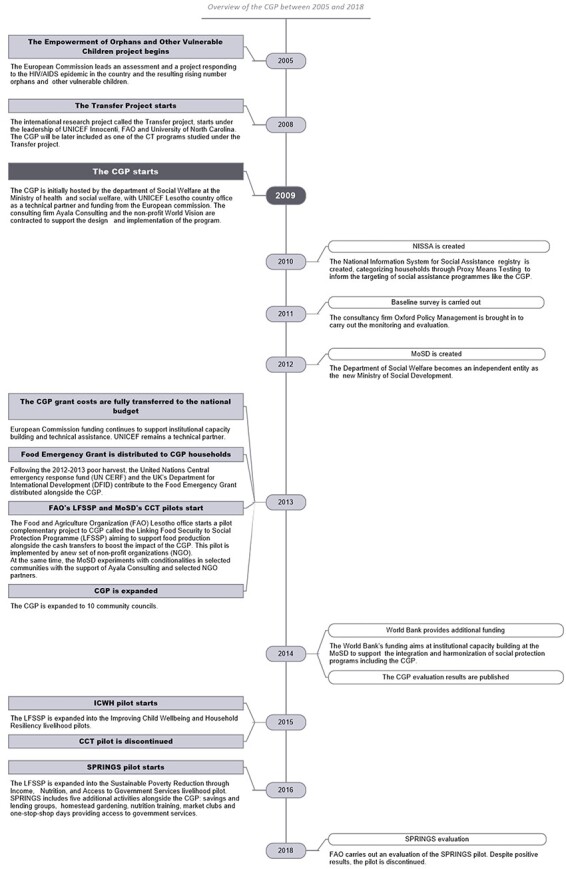
An overview of the CGP between 2005 and 2018

CTs are a key tool in Lesotho’s social protection policy (Granvik, 2016). Initiated after an assessment and pilot led by the European Commission (2005–09), the CGP was first designed as a response to the rising number of OVC caused by the HIV/AIDS epidemic (Pellerano *et al.*, 2016). Figure 2 provides an overview of the CGP (see also Supplementary Annexe 2).

* Pellerano *et al.*, (2014a, 2016), UNC Carolina Population Center, UNICEF Office of Research—Innocenti, Food and Agriculture Organization (2019) and Bhalla (2021).

### Study design

Our descriptive qualitative study relied on the triangulation of information from a review of CGP documents and semi-structured key informant interviews with CGP stakeholders. The thematic analysis facilitated a structured, in-depth examination of the data, allowing for the emergence of critical themes and insights. Data triangulation significantly enhanced the validity of the findings by corroborating evidence from different sources or methods. This methodological approach aimed to ensure a comprehensive understanding and a well-rounded depiction of health equity within the CGP. The detailed description of the method is available in Supplementary Annexe 2.

We focused on the CGP’s early phases (2009–13), before complementary interventions (Cash Plus) were implemented. However, to better understand the evolution of the concepts overtime, we considered elements from the pilot phase (pre-2009) and the post-evaluation phase (post-2014) where relevant.

### Data collection

Data collection was informed by mapping CGP stakeholders using programme documents and consultations with UN agencies in Lesotho with knowledge of economics, politics, gender inequalities, human rights and child health and nutrition in Lesotho.

For the desk review, we carried out a manual search of CGP stakeholder websites and associated programmes’ pages between November 2020 and January 2021 (see Annexe 2, Table SA1). We included 51 programme documents of the 60 documents we screened (see Supplementary Annexe 2, Table SA2).

The sampling strategy for the key informant interviews relied on both purposive and snowballing sampling. Key informants were either:

- professionals directly involved in at least one of the CGP programme cycles [strategic development and programme planning, resource mobilization, implementation, monitoring and evaluation (M&E) and/or research] during all or part of the period of interest (even if that person had moved on to a new post) or
- professionals speaking on behalf of the organizations involved in the programme at the time (referred to as ‘Organizational Point of View’).

To ensure adequate coverage of the different perspectives of the programme, our sampling strategy accounted for the organizations that key informants worked for or represented, their role(s) in the CGP (organizational point of view, team/programme management, operations or analyst/researcher) and the part(s) of the programme cycle they were involved in. We developed a semi-structured interview guide for each stakeholder type. To respect coronavirus disease-infection control guidance, all interviews took place online between June and August 2021. These were audio-recorded, lasting ∼1 h each with 25 key informants from United Nations International Children’s Emergency Fund (UNICEF) entities, the Ministry of Social Development (MoSD), the European Commission Delegation in Lesotho, Oxford Policy Management, Food and Agriculture Organization (FAO), World Vision, Ayala Consulting and the World Bank Lesotho.

After each interview and during the desk review, we wrote short memos to identify potential disagreements or themes and to prioritize questions with individual stakeholders.

### Data coding and analysis

The programme documents and the interview transcripts were coded using NVivo 12.

For the desk review, the coding framework was developed deductively, based on the literature from our conceptual background (Kabeer, 1999; Graham, 2004), the type of document and the programme cycle’s phase covered. To allow more flexibility and the emergence of new themes, we coded the interview transcripts inductively. We also reviewed our memos and documents and applied the NVivo word frequency function on individual transcripts to identify emerging themes. A thematic analysis was conducted. When differences or disagreements arose within a theme, we explored potential determining factors for these variations using additional two-way matrices and charts: we observed the distribution of different points of view across stakeholders’ organizations and characteristics (according to the role and programme cycle); whether informants belonged to an international, national or local team or entity; and the period of the CGP the informants or documents covered. We analysed the documents and interview transcripts separately, before comparing the findings for individual themes.

To ensure the relevance of this study to the CGP, preliminary conclusions and early drafts were shared and discussed with UNICEF Lesotho and the MoSD.

## Results

### Definitions of key concepts

**Figure 3.**
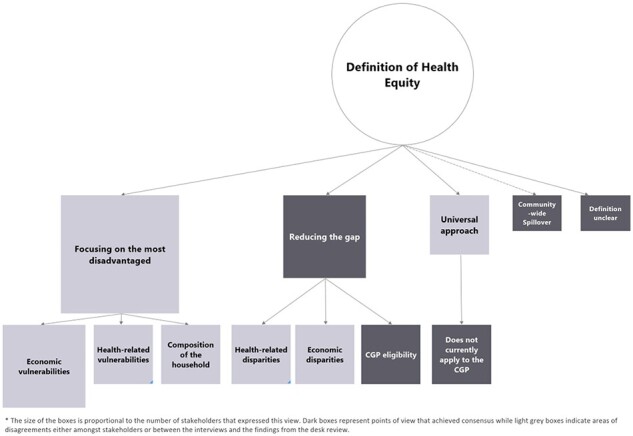
Mapping the definitions of health equity in the CGP

The definition of child health equity varied and was occasionally unclear to stakeholders, reflecting the complexity of such a construct (Figure 3). The most prominent definition of health equity was ensuring ‘access to health services’ for the ‘most disadvantaged children’. Other definitions—‘closing the gap’, ‘universalism’ and ‘spillover’—were seen as minor or not applicable to the CGP. Child health as ‘health status’ or as ‘food security and nutrition outcomes’ were either less common and/or debated (see Supplementary Annexe 3). First, we review the findings of the interviews before comparing to the findings of the desk review.

### Defining health equity

Twenty-three stakeholders discussed the definition of health equity in their interviews. However, five other stakeholders, especially at the lower operational level, raised questions as to what the terminology meant (Box 1).

Box 1  

‘No, I haven’t really seen the notion [of] health equity. Maybe that’s why I’m asking you to unpack it for me because if you do, I can understand. We were not using the term.’

(Implementer, Local)

‘I think people probably did understand different things by it, but it certainly was in the dialogue.’

(Evaluator, International)

Twenty-two stakeholders linked health equity to ‘focusing on the most disadvantaged part of the population’. This focus on health equity as ‘focusing on disadvantage’ most likely reflects the programme’s targeted approach (see From theory to practice: role of health equity in the CGP section). Only two implementers defined health equity as ‘closing a gap between two groups’. A researcher and two implementers linked it to a ‘universal approach’ (Box 2).

Box 2  

‘The objective from the beginning was to alleviate or maybe like close the gap, the health gap that this one shouldn’t be ignored because of their vulnerability.’

(Planner, National)

‘Equity discussions would come in a context where social protection is a bit more mature. When you talk about equity, you’re talking about a social protection platform where the total population in a country can have access to social protection. I think, for now, in Lesotho and in a lot of sub–Saharan African countries, they’re in the stage before where social protection[’s] aim is to enhance extremely poor people’s situation so they can try to exit the cycle of poverty.’

(Implementer, International)

When probed for more detail on these definitions, a greater variety emerges. The definitions linked to a gap or universalism became more broadly reflected. Seven stakeholders referred to a ‘gap’ (a difference between two groups), highlighting primarily health-related and economic disparities between households (Box 3).

Box 3  

‘When we introduced the conditional cash transfer, some were not able to access health services. This means that there was a discrepancy between those who were able to access the services and those who were not. So, when [health equity] came up, I understood it to mean that those were not able to access the services should be at the same level of those who access the services.’

(Implementer, Local)

‘Our perspective is one of equity, of a programme improving equity at the community level, because those people, economically, were left behind.’

(Programme Manager, International)

Five stakeholders referred to elements linked to ‘universalism’ in their definitions—a progressive approach to covering the whole population. However, they suggested that this definition did not apply to the CGP, although it had become part of international stakeholders’ social protection strategy. ‘Community-wide spillovers’ were a common notion (see Role of key concepts, health equity in the CGP) but not necessarily associated with the definition of equity or health equity, with two exceptions (Box 4).

Box 4  

‘Social protection interventions, especially cash transfer programmes that are actually social assistance interventions, are already targeting poor households. You’re already not considering the whole population, but just the bottom part of the distribution in terms of wealth and income. So, I guess that equity was not a priority in the sense that the focus was already on [a] subsample of the population classified as vulnerable, ultra-poor or poor.’

(Researcher/Evaluator, International)

‘The second part of [this concept] is, when households are in the health system, are they able to access health services? By the child grants’ support to the household, then they are able to [provide] those other people with financial support [so that] they are able to access medical care.’

(Organizational Point of View, International)

As the CGP targets poor and vulnerable households, terminology associated with various types of disadvantages was most prominent in informants’ definition of health equity. Twenty-two programme stakeholders identified diverse dimensions of vulnerability and/or poverty, hence the absence of a cohesive list across the interviews (Box 5).

Box 5  

‘Vulnerability is more comprehensive because it’s not only monetary poverty, but also involves food insecurity, lack of access to schools, lack of access to health centers, lack of access to financial institutions to obtain credit or to borrow money. So, when I use the terminology vulnerability, I’m considering a broader concept.’

(Researcher/Evaluator, International)

‘The overall objective of the program is to reduce child poverty. When I say child poverty, it is about reducing deprivation from access to education, primary health care and then also, support families to have proper access to safe water sanitation. So, it’s sort of a combination of four or five indicators’ (Programme Manager, International)

Stakeholders often identified more than one category of vulnerability in their definitions, linking these with contextual factors that led to the development of the CGP: the HIV/AIDS epidemic, poverty (linked to the retrenchment of Basotho workers from South Africa) and food insecurity (linked to recurring adverse climate events). The focus and inclusiveness of these definitions have broadened over time (Box 6).

Box 6  

‘There was a study that was conducted before [the CGP started], which results revealed that most of these children—orphan children—their parents died of HIV and AIDS and most of them came from poor families. It was discovered that they were not attending school properly and food [security] in the house was not good. They couldn’t access the medical services. So, the programme was introduced mainly to lessen the impact of HIV and AIDS. We looked at a household that had no regular income. We looked at a child-headed households. We also looked at households that still had parents, but who were poor, who couldn’t afford to take the children to school or who couldn’t afford to take them for medical services.’

(Implementer, Local)

‘The objective of the Child Grant Programme was to reduce the adverse impacts that poverty, food insecurity, and also HIV and AIDS had on households that were caring for orphans and vulnerable children’

(Evaluator, National)

‘It was very clear that [the programme leaders] wanted to go beyond these orphans or vulnerable children, per definition, and so they defined a target focused on poor households with children’

(Resource Mobilization, International)

In the later phases, a formal list of criteria was developed to guide communities’ definition of vulnerability, although some flexibility remains, according to local specificities (Box 7).

Box 7  

‘For the community-based targeting, we have a guide that is called a wellbeing chart, so the communities will be taken through that wellbeing chart so that they become familiar with the information and the variables. Then, they are allowed and guided into a discussion with the chart. If they feel that in their area, they may want to leave out a particular variable and add one that is missing, they are allowed.’

(Implementer, International)

The term ‘health equity’ was absent from the desk review, but several implications of the concept were identified. As in the interviews, the terminology associated with various types of ‘disadvantages’ was the most prominent in the reviewed documents (Cerritelli, 2009; Hurrell *et al.*, 2011; Ayala Consulting, 2012; Pellerano *et al.*, 2012a; 2014a; 2016; Oxford Policy Management, Andrew Kardan, FAO, 2014). Although no consistent list was found in the programme documents, economic and socioeconomic vulnerabilities were the most common, followed by households’ structure and characteristics (e.g. orphans, child-/elderly-/female-headed households) and health-related vulnerabilities. As M&E documents showed, continuous attention was given to the contextual factors that might fuel these factors of vulnerability (UNICEF Lesotho, 2010; 2017; Hurrell *et al.*, 2011; Kardan *et al.*, 2011; Pellerano *et al.*, 2012b; 2014b; Asfaw *et al.*, 2013; Oxford Policy Management, 2013; Oxford Policy Management, Andrew Kardan, FAO, 2014; Barca *et al.*, 2015). The desk review also put forward more structural factors, such as local market and local economy characteristics, access to services, access to identity documents and institutional support (Hurrell *et al.*, 2011; Oxford Policy Management, 2011; 2013; Kardan *et al.*, 2011; Pellerano *et al.*, 2012b; 2014b; Oxford Policy Management, Andrew Kardan, FAO, 2014; Analysis for Economic Decisions, Analysis for Economic Decisions, 2015).

When referring to a ‘gap’ as a difference between two groups, the reviewed documents mainly distinguished between CGP recipients and non-recipients across social, economic and health dimensions (Hurrell *et al.*, 2011; Pellerano *et al.*, 2012a; 2014a). As for the ‘universal’ approach to equity, the desk review reflected the findings of the interviews (Pellerano *et al.*, 2012a; Analysis for Economic Decisions, Analysis for Economic Decisions, 2015; Islam and Orton, 2019): this approach was not applied to the CGP, despite its growing influence on international stakeholders’ social protection strategy (UNICEF, Overseas Development Institute, 2020).

### From theory to practice: role of health equity in the CGP

**Figure 4.**
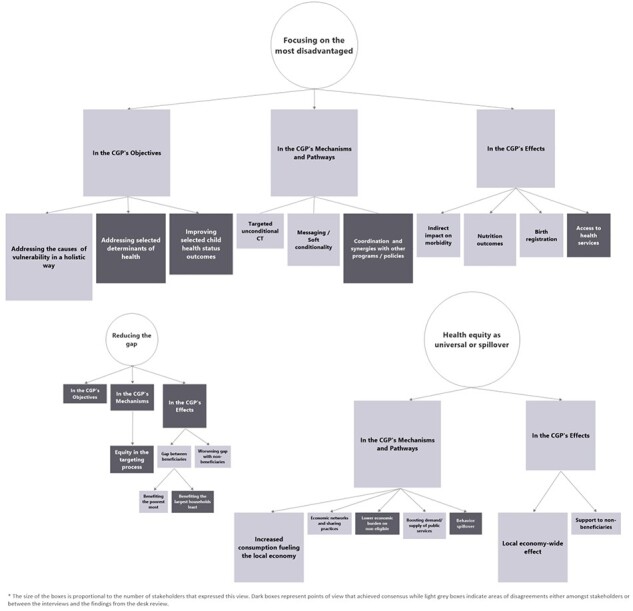
Mapping the role(s) of health equity’s different definitions in the CGP

The diversity of the health equity definitions described above affected how this concept was translated into the CGP (Figure 4). We found that the operationalization of health equity from concept to concrete, observable and measurable applications in the CGP faced many challenges. We first discuss the role of health equity as a general concept, before exploring the role of the three different definitions across the CGP’s objectives, mechanisms of action and effects.

When asked about this concept in general terms, 14 stakeholders, especially implementers, stated that health equity was not an objective of the CGP in the early phases. Ten stakeholders saw health equity as a CGP objective but described it rather as a concern or a strategic goal, which was not translated into operational targets and activities (Box 8).

Box 8  

‘[Health equity] was an overarching goal and something in the back of everyone’s mind. And of course, the broader objective was to achieve this and consider this, but, on the day to day, it wasn’t what was being discussed, it wasn’t what was being considered.’

(Implementer, International)

The document review confirmed this finding. The term ‘equity’ was used in evaluations and research but was widely absent from operational documents (Ayala Consulting, 2012; Thomson and Kardan, 2012; Oxford Policy Management, FAO, 2013; Analysis for Economic Decisions, Analysis for Economic Decisions, 2015; Pellerano *et al.*, 2016; Daidone *et al.*, 2017). However, this finding is nuanced when considering the role of individual definitions of health equity.

#### Health equity as focusing on the most disadvantaged

This definition was deeply ingrained in the programme (Figure 4). However, findings pointed to several operational challenges and to a gap between this definition of health equity as a strategic objective and as an operational target. In this section, we first explore the findings of the interviews and compare these to the findings of the desk review.

Twenty-four stakeholders discussed the role of health equity as ‘focusing on the most disadvantaged’. This definition appeared as a key objective of the programme (*n* = 21), as the CGP aimed to reduce malnutrition, improve health status and increase school enrolment of children identified as vulnerable (Box 9).

Box 9  

‘The CGP was designed and mainly to target the children from poor households and to ensure that they are brought into the mainstream setting of education and health services. (…) We’re mainly focusing on ensuring that the vulnerable children at least meet their daily needs in as far as food is concerned, access to school and access to health.’

(Implementer, International)

To achieve this goal, the CGP seemed to focus on addressing the causes of vulnerability in a holistic way, by investing in these children’s human capital or addressing selected determinants of health, like hygiene, and access to health services. As a comparison, only 11 stakeholders discussed the improvement of specific child health outcomes, primarily nutritional outcomes, as CGP objectives in the early phases (Box 10).

Box 10  

‘For us, CGP is a typical programme to promote human capital(…) In a society, when children go to school, in a long run you’re expecting a society with more educated people, a society where people are contributing, in the economic development sustainable development’

(Programme Manager, International)

‘The CGP came to address those issues: school enrollment was increased because those monies [were] used to buy soap and other cosmetics. The other objective was that whenever children fell sick, the parents would always leave out some small amount of money for transport and to take the child to the health centers.’

(Implementation Manager, Local and national)

‘Secondly, the programme also aimed at reducing malnutrition as well as improving the health status of those children. And also increasing the school enrollment of those children.’ (Implementation Manager, Local and national)

‘I do remember going to schools, I do remember interviewing the teachers and exploring [children’s] education strongly. I have some vague recollections of speaking to health workers, but I don’t remember it being as such pronounced when we were doing our research.’

(Evaluator, International)

This lack of focus on specific health outcomes may also be the result of a divide between the strategic and operational levels. When discussing the focus on the most disadvantaged as an objective of the CGP, implementers in particular described how the overall objective was not translated into operational targets (*n* = 7) or integrated into the programme’s M&E (*n* = 5, Box 11).

Box 11  

‘We wanted to improve health as well as access to health. I can’t really say that they were certain standards or quotas set in terms of what we wanted to achieve besides just mentioning them as overall objectives of the program.’

(Programme Manager, Local and national)

‘I was chasing very operational targets and I’m sure that, in a different room, [programme managers and donors] were talking about outcomes and health targets for example, or education targets, but those were not what I had to report on.’

(Implementer, International)

Focusing on the most disadvantaged was reflected in the mechanisms that the CGP followed and particularly in four programme design features (*n* = 23). First, planners and implementers described adopting a targeted, unconditional CT as a strategic, yet pragmatic, choice to maximize impact (Box 12).

Box 12  

‘I think targeted programmes have had more impact on poverty than universal program.’

(Internal M&E, National)

‘If you select the very poor and come up with this kind of assistance, then, of course, the outcomes will be positive. Therefore, all the donors become interested and, then we can go into more universal kind of benefits.’

(Implementation Manager, International)

As six managers explained, a second mechanism to address health disadvantages was messaging, or soft conditionality, encouraging recipients to attend child health services (immunization in particular) or requiring birth registration to enrol in the CGP (Box 13).

Box 13  

‘It was a soft condition so that, in addition to receiving [the grant], we asked beneficiaries for example to vaccinate their children or to bring them to health centers when they have health issues.’

(Programme Manager, International)

Third, the CGP was coordinated with other organizations’ health and nutrition activities that were designed to holistically address the roots of vulnerabilities (*n* = 6, Box 14).

Box 14  

‘Phase one included distributing seeds to the households. It was a wonderful idea, a holistic approach to all the needs of the family: nutrition, education, food security…’

(Implementer, Local)

Fourth, seven national stakeholders highlighted synergies between the CGP and other government policies and programmes (e.g. free primary care and education; food support). Whether these synergies were in place in the early phases was unclear (Box15).

Box 15  

‘There was a lot of policy discussions on that with the government. But when I left, it was still at the level of discussions. There hadn’t been any decisions taken by the government. The old age pension was not within the realm of the Ministry of Social Development, the old age pension was managed by the Ministry of Finance. (…) You also had another fund that was for public assistance, to really destitute families. Then you also had other kinds of transfers with the Minister of forestry, agriculture. They had some food-for-work or cash-for-work kind of programmes with the World Food Programme, FAO and others. But there was no integration, there was no overarching policy that could make sure that the resources were well distributed’.

(Resource Mobilization, International)

These mechanisms designed to holistically focus on the most disadvantaged were hampered by operational challenges and, particularly, a lack of coordination with other actors. Five stakeholders reported limited involvement of ministries other than the MoSD, and two national stakeholders explained that existing coordination mechanisms did not directly apply to the CGP (Box 16).

Box 16  

‘Since the creation of social development as an [independent] ministry, that has been the missing link between social development and health. We don’t share the reports on whether the beneficiaries of these programmes still attend the clinics as they supposed to. I think that’s the missing link between the two ministries. I think it has to be addressed because I can’t confidently say that we are doing well (…) We don’t specifically have those indicators that are attached to this programme, we don’t report to each other.’

(Implementer, Local)

Some of these challenges were partly addressed in subsequent pilots. Specific child health indicators and monitoring systems were part of the conditional cash transfer (CCT) pilot in collaboration with the relevant ministries. The Sustainable Poverty Reduction through Income, Nutrition, and Access to Government Services (SPRINGS) pilot included further nutrition education and a ‘one-stop shop’ to facilitate access to public services. However, the perceived impact of these later activities on various health outcomes was disputed (Box 17).

Box 17  

‘We implemented a conditional cash transfer, with the intention to try and improve health indicators and education indicators. We mainly wanted to see if we could influence access to health, access to food and to education as well.’

(Programme Manager, Local and National)

‘In the case of SPRINGS, one of the components was to have these outreach days or these one-stop shops, what was in previously called wellbeing days. We found that they weren’t effective. The local government did not have the capacity to really make these one-stop shops operational and did not have the capacity to have these outreach days operational for the most part.’

(Researcher/Evaluator, International)

The desk review’s findings reflected those of the interviews. Improving the health and nutritional status of vulnerable children was the overall objective of the CGP as stated across programme documents (Duynhouwer, 2009; Pellerano *et al.*, 2012b; Oxford Policy Management, 2013). As stakeholders reported, the reviewed documents showed a strong focus on investing in children’s human capital to address vulnerabilities and disadvantage in the long term (Duynhouwer, 2009; UNICEF Lesotho, 2010; 2017; Hurrell *et al.*, 2011; Pellerano *et al.*, 2012b). Although a focus of the evaluation, the improvement of specific health status outcomes for disadvantaged children was widely absent in the CGP’s objectives or anticipated effects in the programme documents (UNICEF Lesotho, 2010; Oxford Policy Management, 2013).

The reviewed documents highlighted the operational challenges the stakeholders discussed. First, M&E documents reflected how cash alone would be insufficient in addressing the barriers to health that stakeholders described (Kardan *et al.*, 2011). Clear, measurable targets for health and nutrition only appeared in the operational documents in relation to the CCT, and a lack of appropriate monitoring of the health objectives was cited as one of the motivations behind the pilot (Ayala Consulting, 2012; 2014, 2015).

### CGP affecting health equity as disadvantage

The impact of the CGP on the health of beneficiary children has already been widely researched and reported in the programme’s evaluations and informants referred to these documents (*n* = 20) (Kardan *et al.*, 2011; Oxford Policy Management, Andrew Kardan, FAO, 2014; Pellerano *et al.*, 2014a; Analysis for Economic Decisions, Analysis for Economic Decisions, 2015; Pace *et al.*, 2019). This included an indirect impact on morbidity, investment in selected determinants of health (e.g. clothes and hygiene), improved birth registration and selected nutrition outcomes. However, stakeholders tended to report more positive CGP effects on access to health care though the evaluations showed mixed findings (Box 18).

Box 18  

‘As far as I can remember, [the CGP] had impact on children’s health but it was not a direct impact. It was something like, if a family can afford warm clothes or shoes for the kids, that protected them from other diseases like coughing, cold. So indirectly, it had impact on health’

(Programme Manager, International)

‘Part of the guidelines of the CGP was that all the children [who] were registered in the programme must have birth certificates. So it also contributed towards increased registration of births, because of that soft condition.’

(Programme Manager, Local and National)

‘The most vulnerable here in Lesotho are found in the mountains. They found that [children] can go days without having a meal or something to eat. With the CGP, that has somehow been affected and impacted positively.’

(Implementer, Local)

‘The CGP contributed to alleviating certain financial barriers to access health care for households that were not able to travel to the health centers. Now that they were receiving the CGP, things have got better because they could afford transport. They also highlight how easy it has become for them to access over-the-counter medicines for their children.’

(Programme Manager, Local and National)

#### Health equity as reducing the gap

Health equity understood as ‘reducing the gap’ played a minor role across the different stages of the CGP in its early phases (Figure 4). Only six stakeholders discussed the role of ‘reducing the gap’ in the CGP, and this terminology was absent from the programme’s documents.

As an objective, stakeholders described it as a broader goal rather than a specific CGP objective (*n* = 2). In the design and implementation of the CGP, three stakeholders defined ‘gap’ as a difference between groups when referring to the targeting of the beneficiaries, to ensure that the programme was effectively enrolling the most vulnerable and poorest households (Box 19).

Box 19  

‘The broader objective of social protection is actually basically social cohesion - broader objective because social imbalance comes from use-inequality between families within the same community. So the purpose of this social protection programme is to reduce inequality, deprivation and enhance social cohesion.’

(Programme Manager, International)

‘Basically we’re saying we don’t really want rich households to benefit. The resources are so scarce, at the very least we just want to make sure that the relatively well-off are not somehow taking advantage. That was the kind of equity concern, I guess.’

(External Evaluator, International)

### CGP affecting health equity as gap

Five stakeholders involved in evaluation or implementation at the local level reported some sporadic effects of the CGP on the health gap, primarily between beneficiaries. The CGP evaluation report also explored gaps between beneficiaries through subgroup analyses (Pellerano *et al.*, 2014a) (Box 20).

Box 20  

‘We might have done some subgroup analysis of whether the poorer households benefited more, which is what you might expect because the cash represents a bigger gain to them’

(Evaluator, International)

‘My personal view is that households are not benefiting equally. If you look at the funding’s design, it’s indexed on the number of children. If you look at that closely, the higher you go in terms of the number of children in the households, [the more] you realize that some children are actually disenfranchised.’

(Implementer, International)

However, one implementer and evaluation document suggested that the effect on the health gap primarily resulted from stagnating or worsening outcomes amongst non-beneficiaries (Box 21) (Pellerano *et al.*, 2014a).

Box 21  

‘[There are] huge gaps between families that are beneficiary and those that are not. The fact that they earn something means that they are better-off than those that are not receiving anything. They are able to access health services now, as compared to those that don’t have anything.’

(Implementer, Local)

#### Health equity as a universal approach or community-wide spillover

Although some international stakeholders understood and promoted health equity as implying a universal approach, this conception of health equity was absent from the CGP in the early phases. Instead, the community-wide perspective in the CGP was addressed through the notion of ‘spillovers’ (Figure 4). Both stakeholders and programme documents stated that community-wide spillovers were not an objective (Box 22) of the CGP but were reflected in some of the programme’s mechanisms of action.

Box 22  

‘I don’t think [the community effect] was part of the design, but as a programmer, you know that there are direct results you are expecting and there could also be some indirect results.’

(Implementer, International)

Twenty stakeholders discussed the programme’s ‘community-wide spillover’ in their interviews. While this definition of health equity was primarily reported by international stakeholders, a wider range of stakeholders saw a role for this definition in the CGP when probed about it. Fifteen stakeholders described spillovers in the CGP mechanisms. The most common mechanism (*n* = 8) was how beneficiaries’ increased consumption fuelled the community’s economy, providing additional revenues to non-beneficiaries producing and selling these supplies. An evaluator and an implementer added that the transfer might lift some of the economic burden placed on non-beneficiaries, thus freeing resources to invest in their own children’s health (Box 23).

Two stakeholders further explained that beneficiaries became able to share and lend resources to other households for them to access child health services and medicines. Second, the entire CGP (including messaging and side activities) and the behavioural change amongst beneficiaries might influence non-beneficiary households (Box 24).

Finally, three stakeholders from international organizations described how CTs can boost the demand for—and thus, stimulate the supply of—public services like health care in the community (Box 25).

The findings from the desk review reflected those of the interviews. In the evaluation documents, spillovers were primarily described in the CGP mechanisms, starting with beneficiaries’ increased consumption boosting local production and sales. These documents also described how such spillovers may occur through the community’s social and sharing networks (Hurrell *et al.*, 2011; Pellerano *et al.*, 2012b). Operational documents provided information on how the CGP could support the development of public and community initiatives (Ayala Consulting, 2010; 2014, 2015; Oxford Policy Management, 2013). However, the desk review did not cover how the CGP may encourage health care seeking or health-related behaviour change amongst non-recipients.

### CGP affecting health equity as spillover

Very few of the effects from these spillover mechanisms were studied in CGP communities and for the most part, did not focus on health effects. According to programme evaluation and eight stakeholders, studies on spillover effects mainly concerned the local economy impact (Local Economy Wide Impact Evaluation—LEWIE) related to the increased consumption, selling and production triggered by the CGP on paydays (Box 26).

Second, both the programme evaluation and two implementers noted positive spillover effects on support given to non-beneficiaries through sharing mechanisms (Box 27) (Pellerano *et al.*, 2014a).

Overall, the evaluation found that spillover effects on other health determinants were minor (e.g. asset ownership, employment, and school enrolment—Box 28) (Pellerano *et al.*, 2014a). The spillover effects on children’s health were mainly presented as potential effects rather than actual effects of the CGP and may have been the result of other policies and interventions implemented at the same time (Box 28).

## Discussion

This descriptive qualitative study explored how stakeholders understood and operationalized the concept of health equity in Lesotho’s CGP. Through interviews and a desk review, we explored how stakeholders involved in the early phases of the programme defined this concept and saw its role(s) in the CGP and the CGP’s effects on health equity.

Box 23  

‘Spillovers were observed from households that were not beneficiaries of this programme because the households that were not that severely vulnerable had some other ways of getting income, through the little things which they were selling. […]. So that exchange means that it also affects the life of that child and this household, which is not that vulnerable.’

(Implementation Manager, Local and National)

‘By giving money to the poorest in a community, you’re relieving a kind of co-responsibility, these informal support mechanisms that would therefore benefit the better-off households in the community because they no longer have to do that [support the poorest].’

(Evaluator, International)

Box 24  

‘Through the child grants support to the household, other people are able to access medical care with [beneficiaries’] financial support.’

(Organizational Point of View, International)

‘I think parents who are not part of the programme are encouraged to send their children to school, encouraged to send their children to routine health checks. Even other households who are not part of this programme would probably be motivated to also send their children to school and to health checks,’

(Planner, National)

Box 25  

‘It had a wider impact also because of the social workers visiting the given community. In a community, if you had maybe 25% of families receiving the child grant programme, somehow there was also an awareness that was there in other families.’

(Resource Mobilization, International)

‘I would also say whenever you have a number of beneficiaries in a small community coming together, then you have greater access to these services than now, and certainly the neighbors will benefit.’

(Organizational Point of View, International)

Box 26  

‘Businesses in the rural areas get much support from these beneficiaries. Where the pay points are, they find that there will be a mini market there during payday and I think it has boosted some sort of local economy and produce.’

(Implementer, Local)

Box 27  

‘Some extended family members [of beneficiaries] reported that they were able to get a soft, soft loan from a recipient household to get medical care. Knowing that your relative has received a grant, you would be able to borrow money or to receive health services.’

(Programme Manager, Local and National)

Box 28  

‘It’s just not enough money to really change things very much, but you’re just making [beneficiaries’] lives a little bit better. So the idea that that this cash injection would be enough to stimulate these broader things that non recipients would benefit from was a bit unrealistic to us.’

(External Evaluator, International)

‘I think the other OVC project components also had a direct impact, maybe because of their approach. They were targeting the entire communities, not segregating by vulnerabilities.’

(Implementation Manager, Local and National)

### Main findings

We highlight three main findings. First, the analysis of health equity definitions in the CGP shows an awareness of health equity issues and their determinants among CGP stakeholders. This is particularly visible in the definition of what constitutes a disadvantage, which was seen as multidimensional and linked to broader contextual factors—a vision that broadened as the programme evolved. However, our definition analysis also reflected the complexity and multidimensional nature of health equity, making it more difficult for stakeholders to articulate a coherent definition and subsequent approach. The term ‘health equity’ was absent from programme documents, while selected stakeholders were unsure of its meaning. *Owusu-Addo et al*. (2019) found a similar lack of recognition and subsequent application for the concept of ‘social determinants of health’ among stakeholders in selected African CT programmes despite their demonstrated impact on such determinants. While our study shows a higher awareness and recognition of health equity and its determinants in the CGP, the meaning of these concepts in the context of CT programmes remains an area for improvement and essential to inform the design and implementation of CTs.

Second, when looking at these definitions individually, our study shows that a majority of CGP stakeholders adopted a narrow definition of child health equity that focused on children’s access to health services for the most disadvantaged households. This definition of health equity suggests that while they understood the impact of broader economic and social factors on health determinants, a majority of CGP stakeholders saw the programme primarily designed to address health determinants at the household level rather than a structural response to the determinants of health inequities. Other definitions of health equity (closing a gap between two groups, universal approach and community-wide spillovers) that may be more inclusive of structural factors shaping health equity were more disputed or seemed to be a terminology primarily used by specific stakeholders. This may reflect the different priorities stakeholders had in the CGP as well as the evolving priorities of selected international organizations in their approach to CT programmes. Looking back at our conceptual background, this suggests that while some international stakeholders are moving further down Graham’s continuum towards a community-wide approach, others remain committed to a narrower, more targeted definition and approach to promoting health equity.

Analysing the role of health equity in the CGP illustrates our third key finding: how operationalization gaps affected the translation of broad health equity strategic objectives into concrete and measurable programme components. In the early phases, the holistic vision of children’s health and wellbeing failed to be translated into operational activities and targets. This ambitious approach was further hampered by operational challenges, such as the difficulty of coordinating several sectors and institutions alongside the CGP. Although some of these gaps were addressed in the subsequent pilots, as of today, these pilots have been discontinued and not yet replaced or integrated into social protection programmes. How individual definitions of health equity were integrated in the different steps of the CGP further reflect this partial operationalization as well as the relative importance of health equity in the programme as compared to other priorities. ‘Focusing on the most disadvantaged’ was the most integrated definition across the programme, confirming its comparative importance with other definitions of health equity. Other dimensions of health equity were brought on ‘not out of’ the CGP but ‘through’ its design features, which may explain why the effects of the CGP on these approaches to health equity were only partially investigated. This may suggest that these definitions were considered less important compared to other mechanisms of the programme but can also further illustrate the operationalization gap, where some of the CGP’s impact pathways were not fully integrated into the M&E system.

### Implication for the CGP and other CT programmes in Africa

Through this study, we can further explore the role of health equity in CT programmes as well as how complex concepts in the international agenda can be translated into action in the field of social protection. Equity or the reduction of unfair inequalities has been integrated into the Sustainable Development Goals 3 and 10 (UN General Assembly, 2015). It is part of UN agencies’ priorities and strategies (UNICEF Executive Board, 2017; Social Determinants of Health, 2021). In Lesotho, equity was part of the government’s Vision 2020 goals on health, gender and education (Government of the Kingdom of Lesotho, 2002). Finally, as the 2023 summit of the Transfer Project ‘Promoting equity and resilience’ illustrates, equity is becoming a rising feature in strategic discussions around CTs among government officials, international development organizations and researchers. Besides the growing use of the term itself, we notice an increased focus on the implications and impact of CT programmes on communities as a whole (beyond beneficiaries alone) and on equity and inclusivity (see, for example, Handa, (2023), Transfer Project (2023) and Winder Rossi (2023)). However, this terminology remains primarily applied to economic or food security objectives and outcomes rather than health issues, for which marginalized and disadvantaged populations remain the focus. In a global context of multidimensional crises fuelling health inequities and as some CT programme stakeholders are increasingly moving towards universal approaches to CTs (Winder Rossi, 2023), the findings from this study can help inform future programme development and design to ensure a better understanding, translation, implementation and evaluation of health equity into CT design.

Health equity remains understudied in the field of social protection (Saran *et al.*, 2020) and is primarily understood as targeting disadvantaged groups. In comparison, although the field of health equity research is not exempt from debates, the terminology itself has been widely adopted by health practitioners and researchers (Kawachi *et al.*, 2002; Commission on Social Determinants of Health, 2008; Saran *et al.*, 2020; Welch *et al.*, 2022). This study confirms that this is not yet the case in the field of social protection. As the equity terminology is rising in CT discourses at the strategic level, our study highlights the need for improving the visibility and shared understanding of such a concept among all CT stakeholders to inform the design and implementation of CTs.

The diversity and divergence of views found in the meaning of health equity and its role in the CGP can also result from stakeholders’ competing and evolving priorities. As CTs are the fastest proliferating type of safety net programme on the African continent (Beegle *et al.*, 2018), lessons from the Lesotho CGP can help foster a more cohesive approach to embedding health equity into these programmes, in a context of the evolving paradigm of social protection. Globally, social protection has shifted from a risk management to a transformative approach that can address the root causes of vulnerability and catalyse wider positive changes in society (Molyneux *et al.*, 2016). Mirroring this evolution, selected international CGP stakeholders seemed to be moving away from focusing on the most disadvantaged and towards a more inclusive or universal approach to child health and wellbeing in CT programmes (as the more minor definitions of health equity showed).

Our study highlights how national and local stakeholders in Lesotho remain focused on the needs of a very vulnerable population, closer to the risk management approach, although they did understand how broader factors were directly affecting these vulnerable groups (Brunori *et al.*, 2010). These stakeholders may not have a clear understanding of the new terminology driving changes at the strategic level or may not share other stakeholders’ new universal approach to health equity. As more CT stakeholders are calling for universal social protection on the African continent and in a global context of competing and changing positions on social protection among development agencies and donors (Devereux and Roelen, 2016; Winder Rossi, 2023), this evolution highlights the importance of building and renewing the consensus amongst stakeholders in CTs. Such a consensus-building exercise will become even more pressing if, as the above-mentioned workshop suggests, equity objectives become more central in international strategic discussions.

Second, our study on health equity highlights the challenges of operationalizing a holistic, multidimensional approach to health equity. Even for the most shared definition of health equity, we found several gaps between the strategic vision and the day-to-day operations and M&E. While consensus building will help address some of the programme operational challenges we have identified, our study also highlights the need for all the strategic elements to be more systematically reflected across programme design features, activities and monitoring targets. Given the constraints of the programme and the Lesotho context, implementation research into the bottlenecks and discrepancies that have led to this operationalization gap would further help ensure the fidelity and impact of the CGP. As the wider policy environment also plays an important role in shaping the design of health equity-promoting CTs (Owusu-Addo *et al.*, 2019), further engagement and collaboration with other sectors outside of social protection in the future evolutions of the CGP can help better integrate health equity consideration into the CGP’s theory of change and design features but also drive the programme’s impact of health equity outcomes.

### Limitations

We made every effort to identify and interview a wide range of informants representing the diversity of stakeholders involved in the CGP’s early phases, thanks to the mediation and support from our focal points in Lesotho. However, staff turnover in selected organizations and stakeholders declining our invitations affected our recruitment. To compensate for these losses, we identified alternate informants either with organizational knowledge or experience in part of the phases of interest for our interviews. We also extended the document search for the desk review, to compensate for potential information gaps.

The reliability of the information provided by informants may have been affected by the recall period of this study: interviews required stakeholders to remember information from over a decade ago. To limit the risk of recall bias, we asked key informants extensive background information on their role and on the chronology of events they referred to in their answers. When relevant, we used references to specific programme documents or wider events to better contextualize the information. Discrepancies and disagreements were explored not only according to the stakeholders’ characteristics but also the chronology of the programme. While the recall period introduces data limitations, it also allowed informants to be more reflexive or even critical of these early phases, thus providing a richer, more transparent view of the programme. The experience of informants since 2014 further supported reflexivity, as many are still involved in similar programmes.

## Conclusion

The CGP was initially designed to target the multifaceted vulnerabilities affecting children amidst prevalent poverty, food insecurity and the HIV/AIDS epidemic. Our study illustrates the initial holistic and ambitious vision of this programme, which explicitly or implicitly touched upon many dimensions of health equity, demonstrating CGP stakeholders’ awareness of the broader determinants of health and health equity. However, the consensus around defining health equity as ‘focusing on the most disadvantaged’ suggests that the CGP followed a narrower, more targeted approach to health equity.

While there were areas of consensus on how this concept was understood, our study found substantial variations in the operationalization of this term between stakeholders and between the strategic and operational levels of the programme. Even the most agreed upon definitions of this concept was not fully translated into the programme, especially in the day-to-day operations and M&E at the local level. Consequently, some potential effects of the CGP, such as its health community-wide spillover, remain understudied, while other effects tied to specific health disadvantages or gaps may have been weakened.

## Supplementary Material

czad116_Supp

## Data Availability

Data will be shared on request to the corresponding author with permission of CGP managers. The interview data underlying this article cannot be shared publicly as the information collected could lead to some of the informants being identified. This data can be shared on reasonable request to the corresponding author. The links to the documents included in the review are available in this article’s reference list. Unpublished documents were provided by their authors/owner by permission.

## Footnotes

1. Theory of change is a methodology designed to support development programmes’ strategic planning and evaluation. Amongst other applications, a theory of change can be used to detail the logical sequence of a programme, describing how individual activities might lead to the desired change or outcomes, and the different pathways though which this change may occur (Vogel, 2012).

## Abbreviations

CGP = Child Grants Programme

CTs = cash transfers

CCT = conditional cash transfer

DFID = Department for International Development

FAO = Food and Agriculture Organization

M&E = Monitoring and evaluation

MoSD = Ministry of Social Development

OVC = orphans and vulnerable children

SPRINGS = Sustainable Poverty Reduction through Income, Nutrition, and Access to Government Services

UN CERF = United Nations Central Emergency Response Fund

## Reflexivity statement

To ensure the relevance of this research to policymakers in Lesotho and a diversity of point of views (especially during analysis), the collaboration behind this article included both national and international researchers and practitioners, selected because of their expertise on Lesotho, CT and/or methodology. The team included both insiders with intimate knowledge and experience of the CGP and outsiders with expertise in the field(s) of interest. The authors’ group includes European, American and African nationals, based in Norway and in Lesotho. This article was led by early-career researchers and practitioners, with support from more experienced professionals. The authors also ensure a review and validation of findings through an internal validation process involving civil servants in charge of the programme of interest, international organizations present in Lesotho and academics with expertise in CTs.
