# Supplementary material for: Exploring health equity in Lesotho’s Child Grants Programme
Source: Health Policy Plan. 2024 Jan 20;39(2):138–55. doi: 10.1093/heapol/czad116 (PMC10883666; doi:10.1093/heapol/czad116)
Supplement: czad116_Supp [file czad116_supp.zip › czad116_Supp/E4HE 2 HE Annex 1_Rev2 clean.docx]

Annex 1. E4HE Lesotho project’s conceptual background

Child health, health equity and the determinants of health

The first few years of life are crucial to the health of the next generation of adults or even that of their children(Wadsworth and Butterworth, 2005; Marmot *et al.*, 2010; Watkins, 2016; Britto, 2017). They are also a key window of opportunity to promote health equity (Heckman, 2008; Field, 2010). There are different ways to define health (in)equity. The term is often used interchangeably with health inequalities (Graham *et al.*, 2006; Whitehead and Dahlgren, 2006). Health inequalities refer to disparities or differences in health outcomes while health inequities are defined as avoidable inequalities (Kawachi *et al.*, 2002; Whitehead and Dahlgren, 2006; Commission on Social Determinants of Health, 2008). Figure 1 highlights key elements of definitions of health equity in the literature. In this case study, we use the general term “health inequalities” when referring to all differences in health (regardless of their cause) and “health inequities” to highlight the un(fairness) and socially-constructed nature of health inequalities. We also try to reflect the exact terminology used by specific authors or by CGP stakeholders.

A key idea behind this concept is that health is shaped by factors around us that are at least partially beyond our control (see Figure 1), arising from the circumstances in which people grow, live, work, and age, and the systems put in place to deal with these issues. These are known as determinants of health, as they shape the health of an individual or a population. These determinants are shaped by wider social, political, or economic factors that define their unequal distributions in society. Because of their avoidable nature, these inequalities are thus considered unfair and unjust, and often referred to as health inequities (definition adapted from (Commission on Social Determinants of Health, 2008; WHO Social Determinants of Health team, 2013). Children under 5 years of age are particularly vulnerable as their health and development rely heavily on their care-givers (Black *et al.*, 2017). Hence, child health is determined both by children’s individual specificities and by their caregivers’ social and environmental circumstances (Commission on Social Determinants of Health, 2008).

Graham’s (2004) typology of interventions illustrates some of the different conceptions and definitions of health inequalities (Figure 1) and how one’s understanding to this concept implies a specific approach to health equity. . At one end of the continuum, health inequalities are defined as an equivalent to health disadvantages resulting from the poverty and exclusion faced by a specific group. Thus, it involves targeted interventions focusing on the needs and vulnerabilities of that group, in order to ensure a minimum health “level” – or floor. As this definition does not take into account the rest of the population, interventions following this approach, while leading to absolute improvements in the targeted group’s health outcomes, may still result in growing disparities between that group and the rest of the population. Under this scenario, a CT program like the CGP would lead to improvement amongst beneficiary children over time, thus reducing their health disadvantage but not necessarily the disparities between beneficiaries and other children in the community.

Approaching health inequalities as the disparities in health between two groups is what Graham defines as the health gaps, which often refer to the difference between the worst off and the better off in a population. While such a definition of health inequalities involves more ambitious targets than that of health disadvantages, it still focuses on disadvantaged groups, whose health should “catch-up” that of the better offs through targeted interventions. Hence, it may ignore the factors and structures that may lead to the privileged position enjoyed by the “better off” group while also ignoring those in-between these two groups. Under this approach, a CT program would make beneficiaries’ health improve faster than the health of other children.

At the end of the spectrum, health inequalities are defined as a gradient, directly related to the socioeconomic structure of a population. Therefore, it involves a population-wide approach that addresses the systemic factors behind such a socioeconomic gradient in order to give each group an “equal chance” to achieve health. This definition does not imply that health in the most privileged groups stagnates or worsens but rather that health in the other groups must improve faster through a comprehensive policy targeting the roots of inequity. This scenario implies that a CT program affects the wider community directly or indirectly and leads to structural changes that makes the whole community healthier and more equitable.

To understand how CT programs might affect child health disparities, it is essential to understand what conception(s) of health equity was adopted in the program and how that concept was put into practice.

Figure 1. Understanding health equity: Mapping elements of definitions


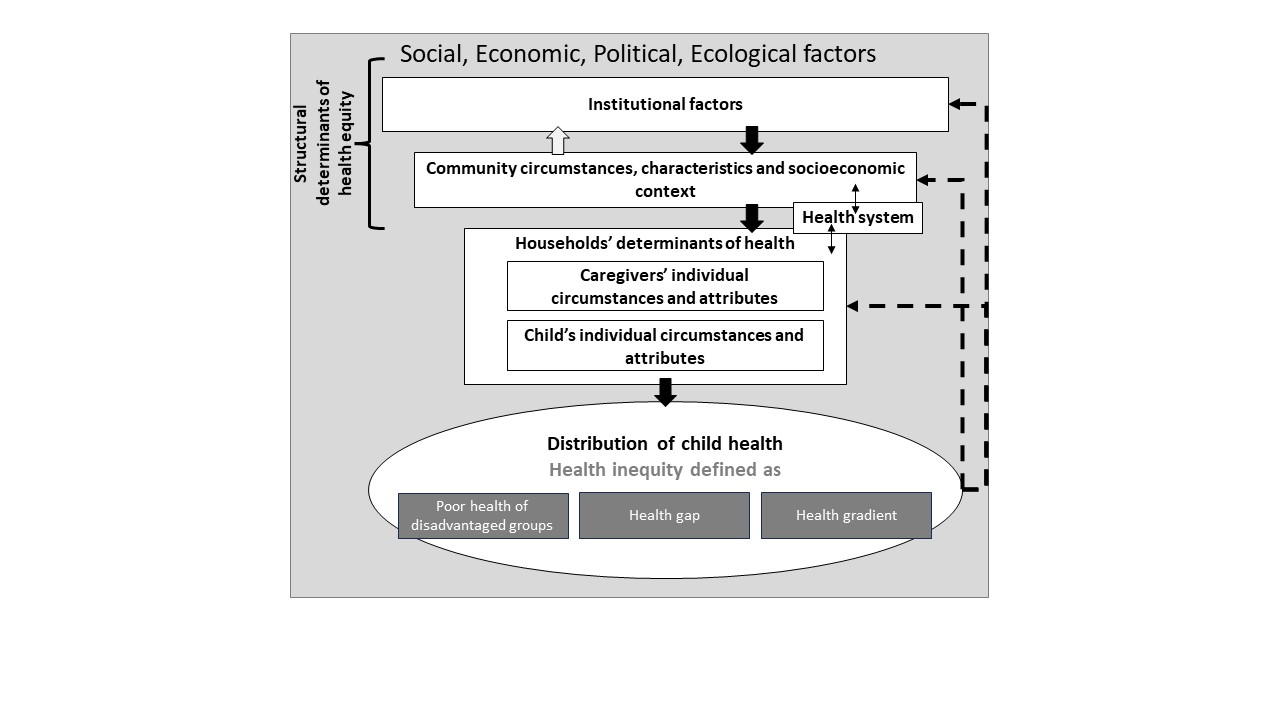


Economic empowerment and health

Power as a determinant of health

The typology presented above highlights how health and health inequalities may be related to the issues of disadvantage or privilege and the factors or structures that create and perpetuate such a gradient. According to the WHO Commission on the Social Determinants of Health (CSDH), power and power relations in society are a key structuring factor in this gradient. Hence, the CSDH considers changing power relations (and the shifting of powers in favor of disadvantaged groups) as a fundamental component of interventions addressing health inequities. Under this approach, health and disease distribution are considered as societally contextualized and health outcomes as socially patterned. While the theoretical literature on the determinants of health and health equity emphasizes different pathways and factors affecting one’s health, all these theories insist on the multiple layers of factors affecting health (see Figure 1) and the crucial role of power and power relations between different groups in these processes (CSDH, 2008). Under this understanding of health, promoting health equity in not only a matter of resources and services but also involves remedying unequal power relations through the empowerment of the comparatively less privileged groups.

Defining empowerment

Several definitions of empowerment can be found in the literature, depending on the type of empowerment discussed and the fields in which it is studied(Narayan-Parker, 2005; Luttrell *et al.*, 2009; Sundström *et al.*, 2017). However, these different definitions do share selected characteristics. The simplest definitions refer to changes in power relationships (Luttrell *et al.*, 2009; Holmes, 2013). More complex definitions describe either a process or an outcome that implies the awareness and capacity to make choices and to act freely on or according to them (agency) to achieve a goal considered desirable (Kabeer, 1999; Luttrell *et al.*, 2009; Donald *et al.*, 2020; GEH, 2020). This can be an individual or collective process (e.g. the empowerment of a group or a community)(WHO, 1998; Keleher, 2009; Luttrell *et al.*, 2009) . Besides individuals’ or groups’ own capacity (the power from within), the notion of freedom is key to empowerment. This directly ties in with enabling and hindering factors that affect one’s power, such as the resources available (and one’s control over them) and the social, economic, political, or cultural context that may promote the power of some over that of others(Kabeer, 1999; Malhotra *et al.*, 2005; Narayan-Parker, 2005; Luttrell *et al.*, 2009; GEH, 2020). In that sense, empowerment is highly context specific (Luttrell *et al.*, 2009). These elements of the definition show the strong influence of A. Sen’s capabilities approach and theory of justice (Holmes, 2013; GEH, 2020). In his theory of justice, A. Sen defends an approach that focuses on equity of capabilities—a person’s ability to pursue a goal they value (Sen, 1995, 2009). As in the definitions of empowerment described earlier, A. Sen sees freedom as a crucial component of capabilities, reflecting the opportunity to pursue one’s objectives but also an absence of constraints or subjugation to the power of others (A. Sen, 2009).

Empowerment can be used as a strategy to improve health in the long run and reduce health disparities or as an outcome of the intervention itself. For example, there is evidence of the health effect of empowering initiatives as public health strategy (WHO, 1998; Wallerstein, 2006; Sen and Östlin, 2008). In the context of cash transfer research, empowerment has been mainly studied as an outcome of these programs for female recipients or members of the households. While there is some evidence that such programs positively affect selected women’s empowerment outcomes (e.g. violence, access to reproductive health, bargaining power), their impact remains unclear and varies according the outcome considered, the specificities of the program or the context in which it is implemented (Bastagli *et al.*, 2016).

At the same time, cash transfer programs themselves can be considered an economic empowerment intervention. By providing additional financial resources, cash transfers can improve households’ access and control over such resources, and their ability to make investment choices, while reducing the effect of shocks or other constraints. As a result, households can invest in social and human capital that can improve their agency (see theory of change in Lesotho’s CGP (Pellerano *et al.*, 2012)).

Women’s economic empowerment and child health

The concept of empowerment is also strongly associated with the feminist movement (Luttrell *et al.*, 2009). Laszlo et al. (2017) defines women’s economic empowerment (WEE) as “the process by which women acquire access to and control over economic resources, opportunities and markets, enabling them to exercise agency and decision-making power to benefit all areas of their lives”. In their categorization of WEE indicators, Laszlo et al. build on Kabeer’s paper (1999) on how to measure WEE. Kabeer identifies four key dimensions of WEE: context (e.g. rights, gender norms or economic opportunities), resources, agency and achievements. Kabeer also highlights the transformative potential of WEE itself and how it can lead to contextual changes such as equal rights and opportunities or new gender norms.

Previous systematic reviews have found that certain markers of women’s social and economic empowerment such as women’s decision-making power and to a lesser extent, economic power or control over economic resources and assets are associated with improved child health outcomes (Duflo, 2012; Richards *et al.*, 2013; Kuruvilla *et al.*, 2014; Carlson *et al.*, 2015; Cunningham *et al.*, 2015; Taukobong *et al.*, 2016; Thorpe *et al.*, 2016).

How the CGP can promote health equity in children

Based on the Child Grant Program’s (CGP) theory of change (Pellerano *et al.*, 2012) and building on the CSDH’s conceptual framework, this unconditional cash transfer program could affect inequalities in child health outcomes and promote health equity in the community both directly and indirectly.

Direct effects

The CGP provides supplementary income to the targeted households, thus reducing poverty and the economic inequalities in the community (Pellerano *et al.*, 2012). Hence it affects targeted households’ socioeconomic position as well as their children’s material circumstances (e.g. clothing, nutrition (Pellerano *et al.*, 2014)). Via these pathways, it contributes to health improvements and may reduce the gap in health outcomes between children in the eligible and non-eligible households (providing that other factors don’t make non-eligible children’s health improve faster). If the theory that female recipients may invest the transfer in more family friendly goods is confirmed than male recipients, this effect should be bigger in female-headed eligible households or households where women are involved in spending decisions.

The CGP may also promote child health equity in the community through another pathway. A previous analysis of program data (Gupta *et al.*, 2018) had found that the CGP led to income spill-over effects for non-eligible households. As a result, by modifying the socio-economic position and material circumstances of other children in the community, the CGP may further affect the structure of health disparities in the treatment communities depending on the health benefit(s) gained by the different groups.

Indirect effects

The CGP may also affect child health and health equity indirectly, through the economic empowerment process it triggers. At the individual level, they can improve recipients’ access to and control over economic resources, their agency and their investment in human development (Barca *et al.*, 2015; UNICEF-ESARO and Transfer Project, 2015; Owusu-Addo *et al.*, 2018). At the household level, CTs can affect the household’s socioeconomic conditions. In turn, this can reduce stress caused by poverty, increase households’ ability to cope with risks and shocks, and affect their emotional wellbeing and intra-household violence or conflicts. Previous literature on CTs also shows that such program can affect power relations and bargaining powers within the household, which can improve both the control over resources and the agency of children’s caregivers (especially women’s) (Slater and Mphale, 2008; Bastagli *et al.*, 2016; Natali *et al.*, 2018; Barrington *et al.*, 2022). At the community level, CTs can support the economic, social and political participation of recipients and place in community’s support and sharing networks, thus strengthening social cohesion (Barca *et al.*, 2015; Molyneux *et al.*, 2016; Owusu-Addo *et al.*, 2018; de Milliano *et al.*, 2021). By affecting these intermediary determinants of health, the CGP may affect the distribution of selected child health outcomes in the community, thus affecting the health gap between beneficiary and non-beneficiary households and/or modifying the structure or gradient of such outcomes.

Empowerment has mainly been studied as an outcome of CT programs for female recipients or members of the households. However, the evidence remains mixed and seems to be highly context-, program- and outcome-dependent (Bastagli *et al.*, 2016; Bonilla *et al.*, 2017, 2017; de Milliano *et al.*, 2021).

Transformative effects

As Kabeer highlights in her research on WEE, economic empowerment interventions also have the potential to positively affect the context in which they are implemented, thus leading to a transformative effect of the intervention. Hence, the CGP – by affecting both health and power structures in the community – could lead to further benefits for child health through the following pathways.

First, by empowering the most vulnerable members of the community, the CGP could potentially lead to structural changes in the community such as: a more equitable income distribution and participation to public decisions, or more equitable norms and opportunities (especially between genders). In turn, these structural modifications would cause further positive changes in the determinants of health and their distribution in the community.

Second, because of their effect on child health itself, CTs like the CGP could lead to a reduction in the community transmission of certain diseases and/or changes in the demand for and access to services within the health system.

While these changes could potentially affect the health gap between eligible and non-eligible, their transformative nature is more likely to be reflected in changes in the health gradient across the whole community, thus leading to a more equitable distribution of child health outcomes, linked with a more equitable distribution of economic power and agency.

Finally, as part of the social protection system, CTs can strengthen the social contract between the State and its citizens and have a transformative impact on power hierarchy and gender norms (Kabeer, 1999; Sabates-Wheeler *et al.*, 2017).

References

Barca V, Brook S, Holland J, Otulana M, Pozarny P. 2015. Qualitative research and analyses of the economic impacts of cash transfer programmes in Sub-Saharan Africa - Synthesis Report. FAO, Rome, Italy.

Barrington C, Peterman A, Akaligaung AJ, Palermo T, de Milliano M, Aborigo RA. 2022. ‘Poverty can break a home’: Exploring mechanisms linking cash plus programming and intimate partner violence in Ghana. *Social Science & Medicine* **292**: 114521.

Bastagli F, Hagen-Zanker J, Harman L, *et al.* 2016. Cash transfers: what does the evidence say? A rigorous review of impacts and the role of design and implementation features. ODI, London.

Black MM, Walker SP, Fernald LCH, *et al.* 2017. Early childhood development coming of age: science through the life course. *The Lancet* **389**: 77–90.

Bonilla J, Zarzur RC, Handa S, *et al.* 2017. Cash for Women’s Empowerment? A Mixed-Methods Evaluation of the Government of Zambia’s Child Grant Program. *World Development* **95**: 55–72.

Britto PR. 2017. Early Moments Matter for Every Child. UNICEF, New York.

Carlson GJ, Kordas K, Murray-Kolb LE. 2015. Associations between women’s autonomy and child nutritional status: a review of the literature. *Maternal & Child Nutrition* **11**: 452–82.

Commission on Social Determinants of Health. 2008. Closing the gap in a generation: Health equity through action on the social determinants of health. WHO, Geneva.

Cunningham K, Ruel M, Ferguson E, Uauy R. 2015. Women’s empowerment and child nutritional status in South Asia: a synthesis of the literature. *Maternal & Child Nutrition* **11**: 1–19.

Donald A, Koolwal G, Annan J, Falb K, Goldstein M. 2020. Measuring Women’s Agency. *Feminist Economics* **26**: 200–26.

Duflo E. 2012. Women Empowerment and Economic Development. *Journal of Economic Literature* **50**: 1051–79.

Field F. 2010. The Foundation Years: preventing poor children becoming poor adults. Cabinet Office, London.

GEH. 2020. A Roadmap for Measuring Agency and Social Norms in Women’s Economic Empowerment. University of California San Diego School of Medicine, San Diego.

Graham H. 2004. Tackling Inequalities in Health in England: Remedying Health Disadvantages, Narrowing Health Gaps or Reducing Health Gradients? *Journal of Social Policy* **33**: 115–31.

Graham ID, Logan J, Harrison MB, *et al.* 2006. Lost in knowledge translation: Time for a map? *Journal of Continuing Education in the Health Professions* **26**: 13–24.

Gupta A, Taylor JE, Davis B, *et al.* 2018. Unequal Cash Transfer Spillovers. *University of California, Davis, Department of Agricultural and Resource Economics Working Paper*: 40.

Heckman J. 2008. The Case for Investing in Disadvantaged Young Children. *CESifo DICE Report* **6**: 3–8.

Holmes R. 2013. *Gender and social protection in the developing world: beyond mothers and safety nets*. Zed Books: London, England.

Kabeer N. 1999. Resources, Agency, Achievements: Reflections on the Measurement of Women’s Empowerment. *Development and Change* **30**: 435–64.

Kawachi I, Subramanian SV, Almeida-Filho N. 2002. A glossary for health inequalities. *Journal of Epidemiology & Community Health* **56**: 647–52.

Keleher H. 2009. Gender Norms and Empowerment: ‘What Works’ to Increase Equity for Women and Girls. In: Sen G, Östlin P (eds). *Gender equity in health: the shifting frontiers of evidence and action*. 1st edition. Routledge: New York, 161–83.

Krieger N. 2011. *Epidemiology and the people’s health: theory and context*. Oxford University Press: Oxford.

Kuruvilla S, Schweitzer J, Bishai D, *et al.* 2014. Success factors for reducing maternal and child mortality. *Bulletin of the World Health Organization* **92**: 533–44.

Laszlo S, Grantham K, Oskay E, Zhang T. 2017. Grappling with the Challenges of Measuring Women’s Economic Empowerment. GrOW Working Paper Series No. GWP-2017-12. Institute for the study of international development, Montreal.

Luttrell C, Quiroz S, Overseas Development Institute. 2009. Understanding and operationalising empowerment. Working Paper No. 308. Overseas Development Institute, London.

Malhotra A, Schuler SR, Boender C. 2005. Measuring Women’s Empowerment as a Variable in International Development. In: Narayan-Parker D (ed). *Measuring empowerment: cross-disciplinary perspectives*. World Bank: Washington, DC, 71–89.

Marmot M, Allen J, Goldblatt P, *et al.* 2010. Fair society, healthy lives: The Marmot Review. UCL Institute of Health Equity, London.

de Milliano M, Barrington C, Angeles G, Gbedemah C. 2021. Crowding-out or crowding-in? Effects of LEAP 1000 unconditional cash transfer program on household and community support among women in rural Ghana. *World Development* **143**: 105466.

Molyneux M, Jones WN, Samuels F. 2016. Can Cash Transfer Programmes Have ‘Transformative’ Effects? *The Journal of Development Studies* **52**: 1087–98.

Narayan-Parker D (ed). 2005. *Measuring empowerment: cross-disciplinary perspectives*. World Bank: Washington, DC.

Natali L, Handa S, Peterman A, Seidenfeld D, Tembo G. 2018. Does money buy happiness? Evidence from an unconditional cash transfer in Zambia. *SSM - Population Health* **4**: 225–35.

Owusu-Addo E, Renzaho AMN, Smith BJ. 2018. The impact of cash transfers on social determinants of health and health inequalities in sub-Saharan Africa: a systematic review. *Health Policy and Planning* **33**: 675–96.

Pellerano L, Hurrell A, Kardan A, *et al.* 2012. CGP Impact Evaluation. Targeting and Baseline Evaluation Report’. Report prepared for the Government of Lesotho. UNICEF-Lesotho, Maseru.

Pellerano L, Moratti M, Jakobsen M, Bajgar M, Barca V. 2014. The Lesotho Child Grants Programme Impact Evaluation: Follow-up Report. UNICEFLesotho (with EU funding and technical support from FAO), Maseru.

Richards E, Theobald S, George A, *et al.* 2013. Going beyond the surface: Gendered intra-household bargaining as a social determinant of child health and nutrition in low and middle income countries. *Social Science & Medicine* **95**: 24–33.

Sabates-Wheeler R, Abdulai A-G, Wilmink N, Groot R de, Spadafora T. 2017. Linking Social Rights to Active Citizenship for the Most Vulnerable: The role of rights and accountability in the ’making and ‘shaping’ of social protection. No. 2017–14. UNICEF Office of Research - Innocenti, Florence.

Sen A. 1995. *Inequality Reexamined*. Harvard University Press.

Sen Amartya. 2009. *The idea of justice*. Allen Lane: London.

Sen G, Östlin P. 2008. Gender inequity in health: why it exists and how we can change it. *Global Public Health* **3**: 1–12.

Slater R, Mphale M. 2008. Cash transfers, gender and generational relations:evidence from a pilot project in Lesotho. World Vision International, Maseru.

Solar O, Irwin A. 2010. A conceptual framework for action on the social determinants of health. Social Determinants of Health Discussion Paper 2 (Policy and Practice) No. 2. WHO, Geneva.

Sundström A, Paxton P, Wang Y-T, Lindberg SI. 2017. Women’s Political Empowerment: A New Global Index, 1900–2012. *World Development* **94**: 321–35.

Taukobong HFG, Kincaid MM, Levy JK, *et al.* 2016. Does addressing gender inequalities and empowering women and girls improve health and development programme outcomes? *Health Policy and Planning* **31**: 1492–514.

Thorpe S, VanderEnde K, Peters C, Bardin L, Yount KM. 2016. The Influence of Women’s Empowerment on Child Immunization Coverage in Low, Lower-Middle, and Upper-Middle Income Countries: A Systematic Review of the Literature. *Maternal and Child Health Journal* **20**: 172–86.

UNICEF-ESARO, Transfer Project. 2015. Social Cash Transfers and Children’s OutcomesA Review of Evidence from Africa. UNICEF Eastern and Southern African Regional Office, Nairobi.

Wadsworth M, Butterworth S. 2005. Early life. In: Marmot M, Wilkinson R (eds). *Social Determinants of Health*. Second Edition. Oxford University Press: Oxford, New York, 31–53.

Wallerstein N. 2006. What is the evidence on effectiveness of empowerment to improve health? WHO EURO, Copenhagen.

Watkins K. 2016. A fair chance for every child. No. 978-92-806-4838–6. UNICEF, New York, NY.

Whitehead M, Dahlgren G. 2006. Concepts and principles for tackling social inequities in health: Levelling up Part 1. WHO Regional Office for Europe, Copenhagen.

WHO. 1998. Health Promotion Glossary. WHO, Geneva.

WHO Social Determinants of Health team. 2013. Social determinants of health: Key concepts.
