# Supplementary material for: Exploring health equity in Lesotho’s Child Grants Programme
Source: Health Policy Plan. 2024 Jan 20;39(2):138–55. doi: 10.1093/heapol/czad116 (PMC10883666; doi:10.1093/heapol/czad116)
Supplement: czad116_Supp [file czad116_supp.zip › czad116_Supp/E4HE 2 HE Annex 2_Rev2 clean.docx]

Annex 2. Method

Study setting

The United Nations Conference on Trade and Development (UNCTAD) has designated Lesotho as a “least developed country” since the creation of the category in 1971 (UNICEF Lesotho, 2019). When the CGP was introduced in 2009, more than half of Lesotho children lived in absolute poverty (i.e. they were deprived in two or more essential dimensions), with rates up to 80% in the mountain areas (UNICEF, 2011). Lesotho had the third highest HIV prevalence rate globally, with an estimated 23% of its adult population living with the virus (Ministry of Health and Social Welfare - MOHSW/Lesotho and ICF Macro, 2010; UNICEF, 2011). The HIV/AIDS epidemic had ripple effects for child health, fueling rising trends in maternal and child mortality in the years prior to the establishment of the CGP (Ministry of Health and Social Welfare - MOHSW/Lesotho and ICF Macro, 2010; UNFPA, 2012). In terms of governance, tensions between political parties, a struggling economy and persistent social and gender inequalities had contributed to recurring political instability (Shale, 2021). Because of Lesotho’s economic dependence on its neighbor, South Africa’s economic crisis in the 1990s and the retrenchment of thousands of Basotho mine workers had lasting impacts on both national and households’ revenues and employment, especially in rural communities heavily dependent on these remittances (UNICEF, 2011; Granvik and UNU-WIDER, 2016; Ministry of Labour and Employment and IOM, 2017). Finally, Lesotho has faced regular extreme weather events, further exacerbating food insecurity (Shale, 2021). As these challenges affected unevenly Basotho communities and households, they contributed to health inequity issues Basotho children (see Table 1 in the manuscript).

Initiated following an assessment and pilot led by the European Commission (2005-2009), the CGP was first designed as a response to the HIV/AIDS epidemic in the country and the resulting rising number of OVC (Pellerano *et al.*, 2016). Initiated following an assessment and pilot led by the European Commission (2005-2009), the CGP was first designed as a response to the HIV/AIDS epidemic in the country and the resulting rising number of OVC (Pellerano et al., 2016). Unlike other CTs in place in Lesotho at the time, the CGP targeted households rather than individuals. From three Community Councils (about 1,250 households) in 2009 the program grew to 10 Community Councils (2,300 households) in 2013 at the time of the first evaluation. The CGP was also expanded from three districts (Qacha's Nek, Mafeteng and Maseru) to five districts (Qacha’s Nek, Maseru, Leribe, Berea and Mafeteng). All these Community Councils were located in rural areas in the lowlands and foothills, with limited access to health services and markets (Pellerano et al., 2014).

Beneficiary households were selected through a mixed method of Proxy Means Testing and community validation. Proxy Means Testing is a census-based targeting designed to identify ultra-poor and very poor households in the community. In Lesotho, the Proxy Means Testing took into account elements such as dwelling conditions, households characteristics and the ownership of selective assets to assess household’s level of poverty (Pellerano et al., 2014a). Since 2010, this process has been run by the National Information System for Social Assistance (NISSA). Community validation relied on local communities or their representatives to identify or review the list of potential beneficiaries, in order to select vulnerable households. The role and importance of communities in the CGP beneficiary selection process as well as the methodology used evolved over time.

As an unconditional CT, the CGP didn’t require beneficiaries to meet pre-defined targets or undertake specific activities to remain in the program. However, the CGP included strong messaging by staff at pay points and by their community that the transfer was to be used for the children (“soft conditionality”). The CGP was initially designed as a randomized controlled trial. The program randomly assigned electoral divisions in each Community Council to a treatment group (where the CGP was distributed to eligible) or a control group. In control areas, eligible households were identified according to the same process as in treatment areas but the implementation of the CGP was delayed until after the first evaluation.

The program was designed as a randomized controlled trial. The baseline survey of the program took place in summer 2011 and the follow-up survey in summer 2013 (Pellerano et al., 2014). The evaluation also included a qualitative analysis of the CGP’s impact on household economy, local economy and social networks; and an economy-wide impact evaluation (LEWIE) assessing the program’s impact on treatment communities’ economy (Oxford Policy Management et al., 2014; Thome et al., 2016).

Study design

Our descriptive qualitative study used thematic analysis and relied on the triangulation of information from a review of CGP documents (e.g. briefs, analyses and reports generated by program stakeholders) and semi-structured key informant interviews with CGP stakeholders. It is one of the qualitative components of the E4HE Lesotho mixed-method case study.

The choice to employ qualitative descriptive research (QDR) stemmed from its profound ability to delve into the 'who,' 'what,' and 'where' of the experiences and events under examination. The straightforward descriptive capacity of QDR serves as a crucial asset in presenting a clear and detailed portrayal of the phenomenon under study.

Additionally, the thematic analysis utilized within this qualitative component facilitated a structured, in-depth examination of the data, allowing for the emergence of critical themes and insights. On the other hand, data triangulation significantly enhanced the validity of the findings by corroborating evidence from different sources or methods. This meticulous methodological approach aimed to ensure a comprehensive understanding and a well-rounded depiction of health equity within the CGP.

Moreover, the rich narrative data yielded from this qualitative descriptive study not only enriches the understanding of health equity but lays a robust foundation for the quantitative components of the mixed-method case study. This inter-method dialogue, where qualitative findings inform and are validated by quantitative data, further underscores the indispensable value of integrating qualitative descriptive research in this research.

We primarily focus on the CGP’s early phases (2009-2013), before complementary interventions (Cash Plus) were implemented alongside the program. However, to better understand the evolution of the concepts overtime, we have also considered elements from the pilot phase (pre-2009) and the post-evaluation phase (post-2014) when relevant.

Data collection

Data collection was first informed by a mapping of CGP stakeholders using program documents. We regularly updated this initial mapping further to the findings of the desk review and contacts provided by key informants. To help contextualize the study, at the beginning of the data collection phase we also consulted UN agencies in Lesotho with knowledge of the country’s economics, politics, gender, human rights, child health and nutrition.

For the desk review, we carried out a manual search of CGP stakeholder websites and associated program’s pages between November 2020 and January 2021 (Table 1): the Transfer project, UNICEF, UNICEF Innocenti, FAO and its “From Protection to Production” project pages, the Government of Lesotho’s MoSD, the University of North Carolina at Chapel Hill’s Carolina Population Center, the European Commission and it Delegation to Lesotho, the UK’s Department for International Development (DFID), United Nations Central Emergency Response Fund (UN CERF), Ayala Consulting, World Vision, Oxford Policy Management, Sechaba Consultants, and Economic Policy Research Institute. We included 51 program documents of the 60 documents we screened. These 51 documents are described in Table 2.

Table 1. Websites searched in the desk review

| Website searched | Search dates (month/day/year) | Websites’ home page |
| --- | --- | --- |
| Transfer project | 11/16/2020 to 11/24/2020 | [transfer.cpc.unc.edu](https://transfer.cpc.unc.edu/) |
| UNICEF | 12/04/2020 | [www.unicef.org](http://www.unicef.org) |
| UNICEF Innocenti | 11/26/2020 to 12/01/2020 | [www.unicef-irc.org](http://www.unicef-irc.org) |
| FAO | 11/26/2020 to 12/09/2020 | [www.fao.org](http://www.fao.org) |
| FAO’s “From Protection to Production” program | 12/04/2020 to 01/06/2021 | [www.fao.org/economic/ptop](http://www.fao.org/economic/ptop) |
| Government of Lesotho’s Ministry of Social Development (MoSD) | 01/06/2021 | [www.gov.ls/ministry-of-social-development](http://www.gov.ls/ministry-of-social-development/) |
| University of North Carolina at Chapel Hill’s Carolina Population Center | 11/28/2020 | [www.cpc.unc.edu](http://www.cpc.unc.edu) |
| European Commission | 11/16/2020 to 11/20/2020 | [ec.europa.eu](https://ec.europa.eu/info/index_en) |
| European Commission’s Delegation to Lesotho | 11/18/2020 | [eeas.europa.eu/delegations/lesotho_en](https://eeas.europa.eu/delegations/lesotho_en) |
| UK’s Department for International Development (DFID) | 11/17/2020 | [devtracker.fcdo.gov.uk](https://devtracker.fcdo.gov.uk/)  [www.gov.uk/government/organisations/foreign-commonwealth-development-office](https://www.gov.uk/government/organisations/foreign-commonwealth-development-office) |
| United Nations Central Emergency Response Fund (UN CERF) | 12/01/2020 | [cerf.un.org](https://cerf.un.org/) |
| Ayala Consulting Corporation | 11/28/2020 | [ayalaconsulting.us](http://ayalaconsulting.us/index.php/en/) |
| World Vision | 1/06/2021 | [www.wvi.org](http://www.wvi.org) |
| Oxford Policy Management | 11/18/2020 | [www.opml.co.uk](http://www.opml.co.uk) |
| Sechaba Consultants | - | website no longer available as of 28/11/2020 |
| Economic Policy Research Institute | 11/18/2020 | [epri.org.za](https://epri.org.za/) |

Table 2. Number of included program documents by type

| Monitoring and evaluation reports | 19 |
| --- | --- |
| Academic papers  (produced by program stakeholders) | 12 |
| CGP manuals  (e.g. operational manuals, M&E guides) | 10 |
| CGP instruments  (e.g. survey questionnaires), | 3 |
| Stakeholder reports  (e.g. annual reports) | 3 |
| Internal briefings | 3 |
| Press release | 1 |
| **Total number of documents included:** | **51** |

The sampling strategy for the key informant interviews with stakeholders relied on both purposive and snowballing sampling. Key informants had to be either

- a stakeholder who was directly involved in the strategic development and program planning, resource mobilization, implementation, monitoring and evaluation and/or research of the CGP during all or part of the time period of interest (including professionals that might have moved on to a new position)
- or a professional able to present the point of view of the organizations involved in the program (referred to as “Organizational Point of View”).

To ensure good coverage of the different perspectives on the program, our sampling strategy took into account the organizations key informants worked for or represented, their role(s) in the CGP (organizational point of view, team/program management, operations, or analyst/researcher) and the part(s) of the program cycle they were involved in (strategic development and program planning, resource mobilization, implementation, monitoring and evaluation (M&E) and/or research). Professionals representing organizations that became involved in the CGP after the period of interest were excluded. We aimed to include at least one person per role in the organization (manager, operational staff, analyst/researcher or informant representing the organizational point of view), and program cycle (strategy development and program planning, resource mobilization, implementation, monitoring and evaluation (M&E) and/or research). We developed the initial list of key informants based on our stakeholder map. Then the list was reviewed and complemented by [the authors institution]. Further contacts and stakeholders were added when relevant as the interviews progressed, based on the information and contacts provided by the key informants themselves. To improve participation, [the authors institution] provided introductions by email or phone and facilitated interview planning with potential informants whenever possible. When a target informant could not be reached or refused to participate, we attempted to secure an alternate contact. Despite these efforts, we were unable to reach or secure interviews with five of our target informants.

We developed a semi-structured interview guide for each type of stakeholder, based on their role in the CGP. To respect infection control guidance in place during the data collection period, all the interviews took place online over about 1 h per informant. To make sure that access to the internet was not a barrier, the team offered to cover the cost of mobile data for the time of the interview if the informant did not have access to a stable institutional connection in Lesotho (however, this did not happen to be the case with any of the interviews). In total, we conducted and audio-recorded interviews with twenty-five key informants from UNICEF entities, the MoSD, the European Commission Delegation in Lesotho, Oxford Policy Management, FAO, World Vision, Ayala consulting and the World Bank Lesotho.

After each interview and during the desk review, we wrote short memos to identify potential disagreements or new themes. These memos were also used to adapt the interview guide and prioritize questions with individual stakeholders. We transcribed and anonymized each interview, using the interview recording and notes. We assigned a code to each informant during data cleaning and coding to ensure anonymity.

Data coding and analysis

The program documents and the interview transcripts were coded using NVivo 12.

For desk review, the coding framework was developed deductively, based on the literature from our conceptual background (Kabeer, 1999; Graham, 2004; Laszlo *et al.*, 2020), the type of document, and the program cycle’s phase covered. To test the suitability of the coding framework and inter-rater reliability between coders, we piloted this coding system with a first batch of five documents that were coded in duplicate. Discrepancies in coding were discussed between the coders and resolved by consensus. After the pilot, the coding framework was revised accordingly. The rest of the documents were split between the two coders, with a sample being cross-checked by the other coder. To discuss and solve discrepancies and challenges in document coding, the coders met regularly throughout the coding process. These meetings also allowed us to exchange questions, thoughts and reflections about the study. These discussions were turned into memos used to inform the interviews and the analysis.

To allow more flexibility and the emergence of new unexpected themes, we coded the interview transcripts inductively. All the transcripts were coded by one coder, with periodic quality checks by a qualitative method specialist to ensure consistency. After the first cycle of coding, we grouped the inductive codes into pre-defined categories similar to those used for the document coding, with some flexibility to allow new categories arising from interviews. As with document coding, we kept notes and memos throughout the coding process to help identify patterns and themes.

A thematic analysis was conducted. First, we reviewed our memos and applied the NVivo word frequency function on individual transcripts or coded sections in the reviewed documents to help identify emerging themes. We also ran two-way matrices on our contextual codes and each code category (e.g. definitions, roles or effects) to better contextualize of the data. When differences or disagreements arose within a theme, we explored potential determining factors for these variations using additional two-way matrices and charts: we observed the distribution of different points of view across stakeholders’ organizations and characteristics (according to role and program cycle); whether informants belonged to an international, national or local team or entity; and the period of the CGP the informants or documents covered. We analyzed the documents and interview transcripts separately, before comparing the findings for individual themes.

Preliminary conclusions and early drafts were shared and discussed with [the authors institution] and the MoSD. These discussions helped ensure the relevance of our findings and conclusions to the Lesotho context and the CGP.

References

Graham H. 2004. Tackling Inequalities in Health in England: Remedying Health Disadvantages, Narrowing Health Gaps or Reducing Health Gradients? *Journal of Social Policy* **33**: 115–31.

Granvik M, UNU-WIDER. 2016. *Policy diffusion, domestic politics and social assistance in Lesotho, 1998–2012*. UNU-WIDER.

Kabeer N. 1999. Resources, Agency, Achievements: Reflections on the Measurement of Women’s Empowerment. *Development and Change* **30**: 435–64.

Laszlo S, Grantham K, Oskay E, Zhang T. 2020. Grappling with the challenges of measuring women’s economic empowerment in intrahousehold settings. *World Development* **132**: 104959.

Ministry of Health and Social Welfare - MOHSW/Lesotho, ICF Macro. 2010. Lesotho Demographic and Health Survey 2009. MOHSW and ICF Macro., Maseru.

Ministry of Labour and Employment, IOM. 2017. Labour Migration Assessment: Lesotho. IOM.

Pellerano L, Daidone S, Davis B, *et al.* 2016. Does Evidence Matter? Role of the Evaluation of the Child Grants Programme in the Consolidation of the Social Protection Sector in Lesotho. In: Davis B (ed). *From evidence to action: the story of cash transfers and impact evaluation in Sub-Saharan Africa*. First edition. Oxford University Press ; Food and Agriculture Organization of the United Nations ; United Nations Children’s Fund: Oxford : [Rome, Italy] : New York, NY, 247–80.

Shale V. 2021. Understanding Conflict, Peace and Gender Context in Lesotho. Government of the Kingdom of Lesotho; UNDP; UN agencies in Lesotho, Maseru.

UNFPA. 2012. UNFPA Country programme document for Lesotho 2013-2017.

UNICEF. 2011. Child Poverty in Lesotho.

UNICEF Lesotho. 2019. Country Office Annual Report 2019. UNICEF-Lesotho, Maseru.
