# Supplementary material for: Exploring health equity in Lesotho’s Child Grants Programme
Source: Health Policy Plan. 2024 Jan 20;39(2):138–55. doi: 10.1093/heapol/czad116 (PMC10883666; doi:10.1093/heapol/czad116)
Supplement: czad116_Supp [file czad116_supp.zip › czad116_Supp/E4HE_2_HE_Annex_3_Rev2 clean.docx]

Annex 4. Defining Child Health

Child health was overwhelmingly defined as *access to health services and medicines* (Figure A3a), while child health as *health status* or as *food security and nutrition outcomes* were either less common and/or debated.

*Figure A3a. Mapping the definitions of child health in the CGP**


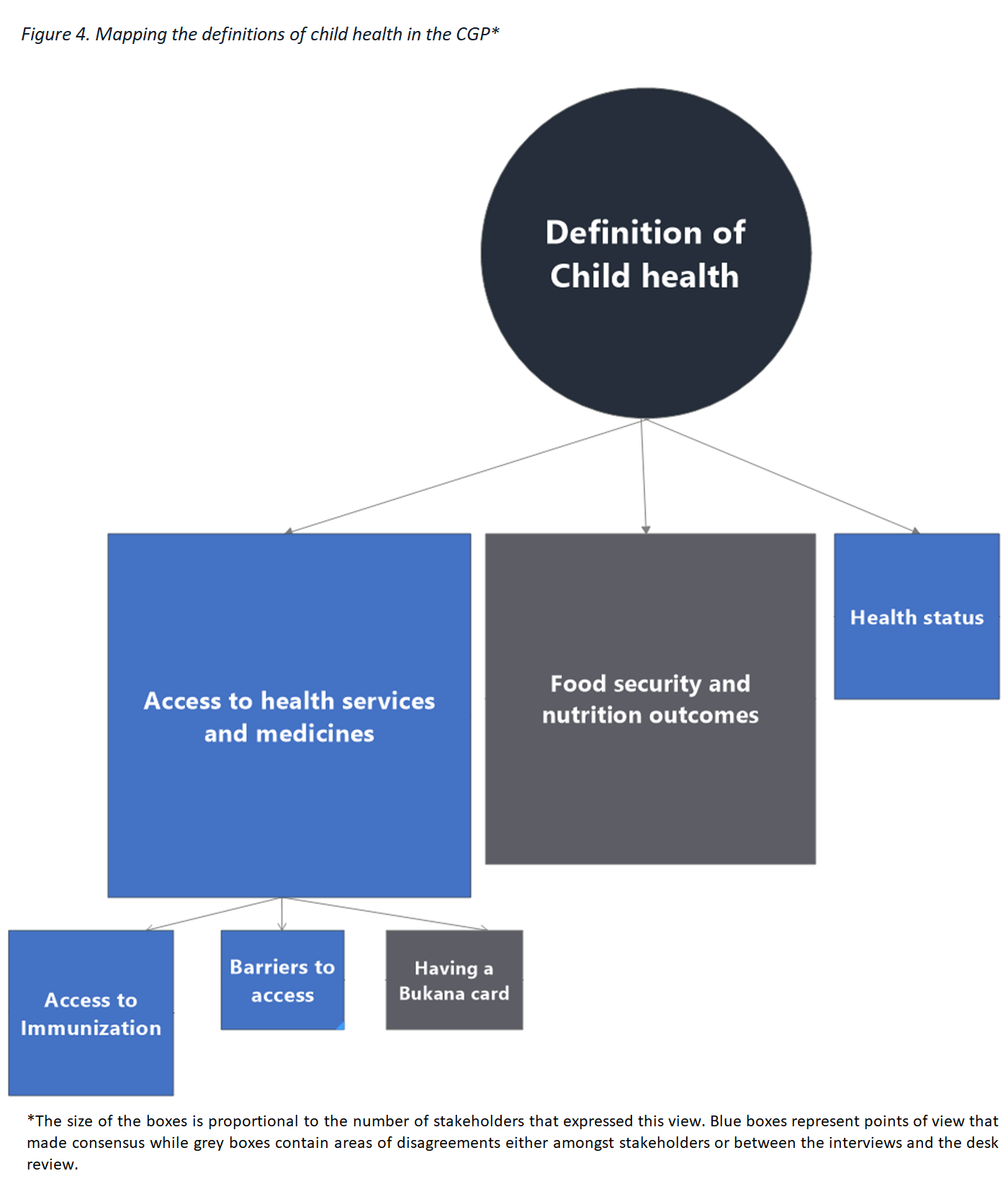


Eighteen stakeholders provided definitions of child health in the context of the CGP. When referring to child health, all but three of these stakeholders referred to *access to health services and medicines*, either in general or regarding specific services, especially immunization. Three stakeholders working at the local and national levels also linked the issue of access to health services with the financial or physical barriers families faced to access child health services in rural Lesotho (Box 1).

Box 1

| “What we knew from the [health objective] was that…Since the health centers are very far of many communities, the patients, whether they are children [or not], struggle to get to the health centers when they are sick. The centers are very far and they don't have transport. So, the messaging was that, when they receive these grants, they should always ensure that there is a little money that is allocated in some cases whenever the children get sick that money is available and used for transport to rush the children to health centers.”  (Implementation Manager, Local and national) |
| --- |

Ten stakeholders also defined *food security and nutrition outcomes* as health. Both of these elements of definitions were found across almost all types of stakeholders regardless of their role in the program (Box 2).

*Box 2*

| “I think the CGP itself had defined the scope as far as specific health outcomes. They were related to immunization, growth monitoring and to a limited extent nutrition. So that is in as far as health was concerned.”  (Implementer, International) |
| --- |

Child health understood as *health status* was far less common and found exclusively amongst five stakeholders in a managerial or leadership position (Box 3).

*Box 3*

| “When we say child health, for us it is about immunization. Then it's about different childhood illnesses - acute respiratory infection, control of diarrheal disease disorders. So there are some basic areas for [our organization] when we say child health”  (Program Manager, International) |
| --- |

As the previous quotes show, stakeholders often included several of these dimensions in their definitions of child health, with access to health services and medicines as the central dimension.

A similar pattern of definitions was found in the desk review, although food security and nutritional outcomes tended to be considered separate outcomes rather than child health outcomes (UNICEF Lesotho, 2010; Hurrell *et al.*, 2011; Kardan *et al.*, 2011; Ayala consulting, 2012; Pellerano *et al.*, 2012a; Oxford Policy Management, 2013; Pellerano *et al.*, 2014b; Analysis for Economic Decisions and Analysis for Economic Decisions, 2015; Food and Agriculture Organization of the United Nations and FAO, 2015; Davis *et al.*, 2016; Pace *et al.*, 2019). The reviewed documents also highlighted barriers to access more prominently when discussing health, as well as the importance of having a Bukana card^^[[1]](#footnote-1)^^ as a proxy for access to healthcare – an element absent from the interviews (Pellerano *et al.*, 2012b, 2014a).

# References

Analysis for Economic Decisions, Analysis for Economic Decisions. 2015. Evaluation of the European Union’s Co-operation with Lesotho 2008-2013 Final report. European Commission, Louvain-la-Neuve.

Ayala consulting. 2012. Technical assistance to the government of Lesotho for capacity building, skills transfers, scale up and transitional arrangements under the Lesotho Child Grants Programme CGP inception report. Government of Lesotho; UNICEF Lesotho, Maseru.

Davis B, Handa S, Hypher N, Winder Rossi N, Winters P, Yablonski J. 2016. Conclusions and Policy Implicationsfor Cash Transfer Programmes. In: *From evidence to action: the story of cash transfers and impact evaluation in Sub-Saharan Africa*. FAO: Oxford : [Rome, Italy] : New York, NY

Food and Agriculture Organization of the United Nations, FAO. 2015. The State of Food and Agriculture 2015: Social Protection and Agriculture - Breaking the Cycle of Rural Poverty. FAO, Rome, Italy.

Hurrell A, Pellerano L, MacAuslan I, Merttens F, Kardan A, Oxford Policy Management. 2011. CGP impact evaluation - Inception report. Oxford Policy Management, Oxford.

Kardan A, MacAuslan I, Merttens F, Pellerano L, Oxford Policy Management. 2011. A Rapid Assessment of the Lesotho Child Grants Programme. Oxford Policy Management, Oxford.

Oxford Policy Management. 2013. Lesotho Child Grant Programme, Follow-Up 2013. Household Questionnaire.

Pace N, Daidone S, Davis B, Pellerano L. 2019. Shaping Cash Transfer Impacts Through ‘Soft-Conditions’: Evidence from Lesotho. *Journal of African Economies* **28**.

Pellerano L, Hurrell A, Kardan A, *et al.* 2012a. CGP Impact Evaluation. Targeting and Baseline Evaluation Report’. Oxford Policy Management, Oxford.

Pellerano L, Hurrell A, Kardan A, *et al.* 2012b. CGP Impact Evaluation. Targeting and Baseline Evaluation Report’. Report prepared for the Government of Lesotho. Oxford Policy Management, Maseru.

Pellerano L, Moratti M, Jakobsen M, Bajgar M, Barca V. 2014a. The Lesotho Child Grants Programme Impact Evaluation: Follow-up Report. UNICEFLesotho (with EU funding and technical support from FAO), Maseru.

Pellerano L, Moratti M, Jakobsen M, Bajgar M, Barca V, UNICEFLesotho (with EU funding and technical support from FAO). 2014b. The Lesotho Child Grants Programme Impact Evaluation: Follow-up Report. Oxford Policy Management, Oxford.

UNICEF Lesotho. 2010. Request for Proposal. Child Grants Programme: Impact evaluation.

1. The Bukana health card is distributed to parents at health care facilities in Lesotho and records key child health indicators - such as weight, height and immunization – for birth until 36 months of age.(Pellerano *et al.*, 2012a) [↑](#footnote-ref-1)
